# Dual transcriptional analysis provides insights into the replicative niche of *P. salmonis* and the host response during infection

**DOI:** 10.1128/msystems.00223-26

**Published:** 2026-04-20

**Authors:** Pamela Aravena, Christian Hodar, Javiera Ortiz-Severín, Khantati Hauyon, Daniel Palma, Mauricio González, Verónica Cambiazo

**Affiliations:** 1 Laboratorio de Bioinformática y Expresión Génica, Instituto de Nutrición y Tecnología de los Alimentos (INTA), Universidad de Chile 14655, Santiago, Chile; 2 Programa de Doctorado en Ciencias Silvoagropecuarias y Veterinarias, Campus Sur, Universidad de Chile 14655, Santiago, Chile; 3 Laboratorio de Bioinformática y Bioestadística Genómica, Instituto de Nutrición y Tecnología de los Alimentos (INTA), Universidad de Chile 14655, Santiago, Chile; 4 Millennium Institute Center for Genome Regulation (CRG), Santiago, Chile; Fluxus Inc., Sunnyvale, California, USA

**Keywords:** *Piscirickettsia salmonis*, dual RNA-seq, lysososmal biogenesis, T4BSS, iron acquisition

## Abstract

*Piscirickettsia salmonis* is a facultative intracellular pathogen and the causative agent of piscirickettsiosis, a systemic disease affecting salmonid fish. Successful replication within host phagocytes is central to disease progression, yet the intracellular niche supporting bacterial growth remains incompletely defined. Here, we employed dual RNA sequencing to characterize the coordinated host and bacterial transcriptional responses during active intracellular replication of *P. salmonis* in SHK-1 cells, a macrophage-like cell line derived from the head kidney of *Salmo salar*. Infection was associated with the induction of lysosome-related pathways in host cells, including increased expression of genes involved in lysosomal biogenesis and proteolytic activity. Concomitantly, *P. salmonis* upregulated genes associated with intracellular adaptation, including components of the Dot/Icm type IVB secretion system, stress-response pathways, and iron-acquisition systems. Confocal microscopy revealed that bacteria reside within an acidified, Lamp-1–positive vacuole resembling a late endosomal/lysosomal compartment. Pharmacological disruption of vacuolar acidification significantly reduced intracellular *P. salmonis* replication, and iron supplementation partially restored growth under conditions of impaired acidification. In line with this observation, experimental manipulation of host iron availability modulated intracellular bacterial growth. Finally, bacteria recovered from infected cells exhibit enhanced replication during subsequent infections, indicating that intracellular passage influences bacterial physiological state. Together, these findings delineate key physiological features of the intracellular niche that shape *P. salmonis* replication and provide a framework for understanding host–pathogen interactions during infection.

**IMPORTANCE:**

Successful intracellular replication is a defining feature of many bacterial pathogens and directly influences disease outcome. For the salmonid pathogen *Piscirickettsia salmonis*, the intracellular environment that supports bacterial growth has remained incompletely characterized. Here, we show that *P. salmonis* replicates within an acidified, Lamp-1–positive vacuole and that intracellular growth is influenced by host iron availability. Infection is accompanied by activation of lysosomal pathways in host cells and coordinated induction of bacterial stress-response mechanisms, secretion systems, iron-acquisition pathways, and numerous genes of previously unknown function. Intracellular passage also alters bacterial behavior during subsequent infection cycles, suggesting a physiological adaptation associated with host-cell residence. By defining the intracellular context in which *P. salmonis* proliferates and situating these features within the broader landscape of intracellular bacterial strategies, this work advances understanding of host–pathogen interactions in non-mammalian systems and provides a foundation for future functional studies relevant to aquaculture and intracellular microbiology.

## INTRODUCTION

*Piscirickettsia salmonis* is the etiological agent of piscirickettsiosis, also known as Salmon Rickettsial Septicemia (SRS), a systemic disease that has an impact on salmonid aquaculture in Chile (1). SRS predominantly affects fish during the seawater stage of production and accounts for approximately 50% of infectious disease-associated mortality in Atlantic salmon (*Salmo salar*) (2). *P. salmonis* is a gram-negative facultative intracellular bacterium capable of surviving and replicating within macrophages in a membrane-bound vacuolar niche (3–5). The bacterium is transmitted horizontally through the gills and skin of fish, even in the absence of physical contact, and triggers a systemic infection characterized by the colonization of various fish organs, including the kidney, liver, spleen, intestine, brain, and gills (6).

Genome sequencing of the reference strain LF-89 revealed genes encoding components of the Dot/Icm type IVB secretion system (T4BSS) and iron-acquisition pathways, among other factors potentially linked to virulence (7). Subsequent transcriptomic and proteomic studies have documented differential expression of genes associated with exotoxins (8), secretion systems, flagella, iron uptake, and amino acid metabolism under diverse *in vitro* and *in vivo* conditions (5, 9–13). Despite these advances, the regulatory mechanisms governing intracellular adaptation and replication of *P. salmonis* remain incompletely understood.

A defining characteristic of *P. salmonis* is its capacity to persist within professional phagocytes, where it establishes a *P. salmonis*–containing vacuole (PCV) (3, 5). As observed with other intracellular pathogens, prolonged residence within host phagocytes likely imposes selective pressures that favor bacterial strategies to tolerate antimicrobial responses and modulate innate immune pathways (14–16). In previous work, we used a microarray-based approach to examine the transcriptional profile of *P. salmonis* at late stages of infection (12 days post-infection [dpi]) in SHK-1 cells, a macrophage-like cell line derived from Atlantic salmon head kidney (17). At that stage, *P. salmonis* displayed a transcriptional pattern resembling stationary-phase growth in broth, marked by reduced expression of translation and replication genes and increased expression of virulence- and stringent response-associated genes (5), suggesting physiological adaptation to prolonged intracellular stress (18).

However, the transcriptional landscape of both the host and the pathogen during the early stages of infection, when *P. salmonis* is actively replicating within host endosomal compartments, has not been comprehensively examined. Understanding the coordinated responses of host and pathogen during this replicative phase is essential to define the intracellular environment that supports bacterial growth. Previous studies have documented host responses to *P. salmonis* infection, including induction of proinflammatory cytokines, oxidative stress pathways, and genes involved in metabolism and intracellular trafficking (13, 19–22). Nevertheless, a comprehensive understanding of the interaction between the host and bacterial transcriptional programs during active intracellular replication is still lacking.

In the present study, we applied dual RNA sequencing (RNA-seq) to simultaneously profile host and pathogen transcriptomes during infection of SHK-1 cells with *P. salmonis* strain CGR02. This standardized infection method, which was developed in previous works (5, 11, 23), enables discrimination between early and late infection stages. Our objective was to define the host cellular environment accompanying bacterial replication and to characterize bacterial gene expression programs engaged within this niche. By integrating transcriptional analyses with functional perturbation of host pathways, we identify key physiological features of the intracellular context and highlight bacterial processes associated with adaptation to this environment. These findings provide a conceptual foundation for future mechanistic studies of *P. salmonis* pathogenesis.

## MATERIALS AND METHODS

### Cells and bacterial culture conditions

SHK-1 cells were obtained from the European Collection of Authenticated Cell Cultures (ECACC N° 97111106) and were cultivated at 18°C in L-15 Medium (Leibovitz, Gibco, USA) supplemented with 5% of inactivated fetal bovine serum (FBS) and 40 μM of 2-mercaptoethanol. *P. salmonis* CGR02 strain (GCA_001534725.1) used in this study was isolated from *S. salar* liver, and it was obtained from ETECMA E.I.R.L (2016, Puerto Montt, Chile).

### *P. salmonis* growth conditions and purity testing

*P. salmonis* was routinely grown at 180 rpm and 18°C on Nutrient *Piscirickettsia* Broth (NPB) until it reached the exponential or stationary growth phase before harvesting for infection experiments or RNA purification. Purity of *P. salmonis* cultures was assessed by a PCR-RFLP assay previously described in Mandakovic et al. (24). A step-by-step protocol is available on protocols.io: dx.doi.org/10.17504/protocols.io.j8nlkrqz1v5r/v1.

To evaluate the replication of *P. salmonis* at different pH levels, a defined medium (CMMAB [12]) was used. Growth at different pH values was assessed as previously described (25), with some modifications. Stationary-phase cultures (1 × 10⁷ bacteria) were inoculated into CMMAB medium adjusted to pH 4.0–6.2. Optical density at 600 nm (OD_600_) was measured every 12 h, reaching 0.5–0.6 after 50 h under optimal conditions. At this time point, samples from all pH conditions were collected for DNA extraction and genome-equivalent quantification as described below. Fold change in genome equivalents was calculated relative to the initial inoculum. Each condition was analyzed using three biological and three technical replicates.

### *In vitro* infection and re-infection of SHK-1 cells

SHK-1 cells were seeded at 3.5 × 10⁵ cells per T-25 flask or 1.5 × 10⁶ cells per T-75 flask in Leibovitz’s L-15 medium supplemented with 5% fetal bovine serum. After 24 h, cells were infected with *P. salmonis* CGR02 at a multiplicity of infection (MOI) of 100:1 (bacteria:cell) and incubated at 18°C. At 3 dpi, extracellular bacteria were eliminated by treatment with gentamicin (100 µg/mL) for 40 min. Infection progression was monitored by inverted light microscopy. A step-by-step protocol for *in vitro* infection and the determination of the percentage of *P. salmonis* containing vacuoles (PCVs) is available on protocols.io: dx.doi.org/10.17504/protocols.io.j8nlkr qz1v5r/v1.

With regard to the re-infection assays, the recovery protocol for intracellular *P. salmonis* and the most probable number (MPN) methodology used to evaluate the viability of recovered bacteria are described in: indx.doi.org/10.17504/protocols.io.261ge8zoog47/v1.

### Genome equivalents of intracellular *P. salmonis*

SHK-1 cells were infected with *P. salmonis* as described above. At selected time points, monolayers were treated with gentamicin (100 µg/mL), followed by incubation with 0.25% trypsin-EDTA for 30 min at room temperature. Cells were recovered by centrifugation, and genomic DNA was extracted from three independent infected cultures per time point using the Blood & Tissue kit (Qiagen, USA). Genome equivalents were determined by qPCR using TaqMan chemistry targeting the single-copy *recF* gene, as detailed in the protocol available at protocols.io: dx.doi.org/10.17504/protocols.io.36wgqq82ogk5/v1.

To assess intracellular bacterial load under iron-modulated conditions, SHK-1 cells were seeded in six-well plates at 50% confluence (1.8 × 10⁵ cells/well) and subjected to one of two treatments: (i) cells were pretreated with 25 µM iron(III) nitrilotriacetate (Fe-NTA) for 4 days prior to infection and collected at 3, 5, and 9 dpi or (ii) cells were infected and monitored daily by bright-field microscopy, followed by the addition of 100 µM deferiprone (DFP) at 7 dpi and collection at 7, 8, and 11 dpi. In both protocols, infections included live or heat-inactivated bacteria as controls. Genomic DNA was extracted as above, and genome equivalents were quantified by qPCR targeting the single-copy *glyA* gene as previously described (5).

### Cell viability assays

Cell viability was quantified using the non-toxic reagent alamarBlue (Thermo Fisher Scientific) as described in reference 5, except that fluorescence of the plates was measured with an excitation filter at 520 nm and an emission filter at 580–640 nm in a GloMax Explorer Multimode Microplate Reader (Promega). Percentages of alamarBlue reagent reduction were calculated with respect to cells infected with heat-inactivated bacteria, following the formula of Al-Nasity et al. (26).

### RNA extraction procedures

Monolayers from nine T25 flasks of infected or uninfected SHK-1 cells were washed twice with PBS and incubated on ice for 30 min in lysis buffer (0.1% SDS, 1% acidic phenol, and 19% ethanol in RNase-free water) as described previously (27). Cells were scraped, and lysates from three flasks were pooled to generate three biological replicates. Samples were centrifuged at 10,000 × *g* for 30 min at 4°C, and pellets were resuspended in 1 mL TRIzol Reagent (Invitrogen). Total RNA was purified using the RiboPure-Bacteria kit (Thermo Fisher Scientific) according to the manufacturer’s instructions. For bacterial RNA extraction, 1 mL of three independent *P. salmonis* liquid cultures was centrifuged at 8,000 × *g* for 15 min, and pellets were resuspended in 1 mL TRIzol. RNA purification was performed according to the manufacturer’s protocol. In all cases, RNA pellets were resuspended in 40 µL RNA Secure (Thermo Fisher Scientific), incubated at 60°C for 15 min, and treated with Turbo DNA-free (Invitrogen). RNA quantity was measured using a Qubit Fluorometer (Life Technologies), and integrity was assessed with a 2200 TapeStation (Agilent Technologies).

### RNA sequencing and analysis

Three RNA-seq libraries from *P. salmonis*-infected SHK-1 cells were generated at the Duke Center for Genomics and Computational Biology using the TruSeq Stranded Total RNA Library Prep kit (Illumina) with eukaryotic rRNA removal (Ribo-Zero Gold). Libraries were sequenced (150 bp paired-end) on an Illumina NovaSeq 6000 across two lanes. Additionally, three libraries from exponentially growing *P. salmonis* in NPB and three from uninfected SHK-1 cells were prepared using TruSeq Stranded Total RNA Library Prep with the Illumina Ribo-Zero Plus rRNA Depletion Kit and sequenced (150 bp paired-end) on a NovaSeq 6000 platform (Novogene Corporation Inc., Durham, NC).

Raw reads were quality-checked with FastQC (28), and low-quality reads and adapters were removed using BBduk (29). Filtered reads were mapped (Table S1) to the *P. salmonis* CGR02 genome (GenBank GCF_001534725.1) and the *Salmo salar* genome (GenBank GCA_905237065.2) using STAR (30). Reads mapping to multiple loci or failing to map were excluded. Gene-level counts were generated using STAR (quantMode). Count matrices were filtered to remove genes expressed in only one condition or in fewer than two of three replicates per condition, and low-abundance genes were excluded following Chen (31). Two comparisons were performed: (i) infected versus uninfected SHK-1 cells and (ii) intracellular versus NPB-grown *P. salmonis* (Fig. S1). Normalization was performed using the TMM method (32), counts were transformed using voom (33), and differential expression was assessed using an empirical Bayes approach implemented in R v4.3.1 with limma v3.56.2 and edgeR v3.42.4. Visualizations were generated using EnhancedVolcano (v1.20.0), ComplexHeatmap (v2.18.0), and ggplot2 (v3.4.4).

### Bioinformatics analysis of transcriptomes

For SHK-1 cells, Gene Ontology (GO) and KEGG annotations were compiled using NCBI and ENSEMBL annotations for the *S. salar* genome (v3.1) and a local annotation generated with eggNOG-mapper v2 (34). *P. salmonis* CGR02 proteins were annotated using eggNOG-mapper v2.1.9 and KofamScan v1.3.0 with the KOfam database (35). In both cases, custom org.db packages were generated using AnnotationForge v1.42.2 (R). GO and KEGG enrichment analyses were performed with the enrichGO function from clusterProfiler (v4.8.1). Heatmaps were generated from normalized expression data expressed as z-scores.

Homologs of Virulence Factor Database (VFDB) proteins (36) were identified using DIAMOND v2.0.15 (37). Putative T4BSS effectors were predicted using a multi-tool strategy integrating Bastion4 (38), CNN-T4SE PSSM (39), T4Sepp (40), and T4SEfinder (41), which apply supervised machine-learning models trained on experimentally validated effectors and incorporate sequence composition, physicochemical properties, and evolutionary features. To increase robustness and minimize false positives, only candidates identified by at least three independent tools were retained.

### Quantitative real-time PCR assays

Real-time qPCRs were carried out in an AriaMx Pro thermal cycler (Agilent Technologies). cDNAs were synthesized from 1 µg of RNA using the High-Capacity RNA-to-cDNA Kit (Applied Biosystems) according to the manufacturer’s instructions. PCR conditions were 95°C for 5 min followed by 95°C for 15 s, 57°C–60°C for 15 s, and 72°C for 20 s for a total of 35 cycles. Melting curves ensured that a single product was amplified in each reaction. Pfaffl method (42) was used to determine relative gene expression levels, using *P. salmonis recF* gene or *S. salar* elongation factor 1α (*elf1α*) as internal reference genes (23). At least three biological replicates were analyzed, and PCR efficiencies were determined by linear regression analysis performed directly on the sample data using LinRegPCR (43). Table S2 provides the complete list of primers used in this study.

### Indirect immunofluorescence assays

SHK-1 cells were seeded on Thermanox coverslips (Invitrogen), fixed with 4% paraformaldehyde (25 min), permeabilized with 0.1% saponin in PBS, and blocked with 0.1% saponin plus 3% BSA for 45 min. Cells were incubated for 1 h at room temperature with anti-Lamp-1 (ab24170, Abcam; 1:200) and anti-*P*. *salmonis* (Ango, clone 7G4/D9; 1:500), followed by anti-mouse Cy5 and anti-rabbit Cy3 secondary antibodies (1:200, 1 h). Additional staining included Alexa Fluor 647 phalloidin, FM1-43Fx, and DAPI (1:200; Thermo Fisher Scientific). For the detection of acidic compartments, cells were incubated with 100 nM LysoTracker Red DND-99 (1.5 h, room temperature; Thermo Fisher Scientific), fixed with 4% paraformaldehyde for 25 min, and processed for immunofluorescence. Images were acquired using a Nikon C2+ confocal microscope with NIS-Elements software. At 7 dpi, the proportion of *P. salmonis*-positive vacuoles co-labeled with Lamp-1 or LysoTracker was quantified from confocal images of at least 80 infected cells obtained in two independent experiments.

### Labeling of acidic and proteolytic organelles

Infected and uninfected SHK-1 cells were incubated for 1.5 h at 18°C with 100 nM LysoTracker red DND-99 or with Magic Red Cathepsin B Assay Kit (ImmunoChemistry Technologies) at a 1:25 dilution in L-15 medium without FBS supplementation. Then, cells were washed with PBS and incubated with Hoechst 33,342 (Thermo Fisher Scientific) for 20 min. Cell images were acquired in a C2+ Confocal microscope (Nikon) using the NIS elements program (Nikon), and the CY3 fluorescence gain value (HV) was set to 110 and the background value to 0. The fluorescent area of each cell was demarcated using the “Binary toolbar” tool of the software, and then the “Measurement result tool” was used to quantify the average fluorescence of the selected area. Fluorescence was expressed as arbitrary units (AU), with the maximum fluorescence value being 4,000 AU. At least 90 cells were analyzed per condition.

### Western blotting

Monolayers from nine T25 flasks of infected or uninfected SHK-1 cells were lysed at 7 dpi using RIPA buffer supplemented with Complete Protease Inhibitor (Roche) for 20 min on ice. Lysates from three flasks were pooled to generate three biological replicates per condition, centrifuged at 10,000 × *g* for 15 min at 4°C, and protein concentration was determined using the Pierce BCA assay (Thermo Fisher Scientific). Proteins were resolved in a 10% SDS-PAGE and transferred to polyvinylidene difluoride (PVDF) membrane overnight at 4°C. Membranes were blocked in Tris-buffered saline, 0.05% Tween 20, and 5% skim milk (TBST). Membrane was incubated with anti-Lamp-1 (1:30,00; PA1-654A, Invitrogen) and anti-β-tubulin E7s (1:3,000; DSHB Cat# E7, RRID:AB_528499) overnight at 4°C and then with anti-rabbit horse-radish peroxidase (HRP) (1:5,000; Cell Signaling) or anti-mouse HRP (1:5,000; Cell Signaling) for 1.5 h at room temperature. Blots were revealed using SuperSignal ELISA Femto Maximum Sensitive Substrate (Thermo Scientific) and exposed on a C-DiGit Blot Scanner (LicorBio). Immunoblots were analyzed using Image Lab Software, version 6.1.0 (BioRad Laboratories).

### Effect of Baf A1 or CQ on SHK-1 cells and *P. salmonis*

SHK-1 cells were seeded in 96-well plates at 1 × 10⁴ cells/well (*N* = 12 wells per treatment) and treated for 24 h at 18°C with 250 nM bafilomycin A1 (BafA1), 25 µM chloroquine (CQ), or 0.75 µL DMSO (control). Cells were then washed with PBS and incubated in fresh L-15 medium supplemented with 5% FBS for 7 days. Cell viability was assessed as described above. To evaluate potential direct effects on bacterial growth, NPB medium was supplemented with 250 nM BafA1, 25 µM CQ, or DMSO and inoculated with *P. salmonis* at an initial OD_₆₀₀_ = 0.03. Bacterial growth was monitored daily by OD_₆₀₀_ measurements for 5 days. To assess the impact of BafA1 on SHK-1 phagocytic capacity, cells were seeded on Thermanox coverslips (6 × 10⁴ cells/well), treated with 250 nM BafA1 for 24 h, and incubated for an additional 24 h with fluorescent beads (FluoSpheres yellow-green, 2 µm; Thermo Fisher Scientific) at a 1:10 cell-to-bead ratio. Cells were washed with 0.3 M glucose in PBS, fixed with 4% paraformaldehyde for 25 min, and stained with Hoechst 33342 and WGA-633 (Thermo Fisher Scientific). Images were acquired using a Nikon C2+ confocal microscope and analyzed with NIS-Elements software. Phagocytic capacity was quantified as the percentage of cells containing FluoSpheres per field of view, and the number of internalized beads per cell was also recorded.

### BafA1 and CQ treatments of SHK-1 infected cells

SHK-1 cells were treated with 250 nM BafA1, 25 µM CQ, or 0.75 µL of DMSO for 24 h, washed with cold PBS, and then infected with *P. salmonis*. Additionally, cells were treated with 25 µM Chloroquine or 0.75 µL of DMSO for 24 h, washed with cold PBS, and then supplemented or not with 25 µM Fe-NTA for 3 days during *P. salmonis* infection. Genome equivalents were measured at 3 and 7 dpi as described above.

### Preparation of Fe-NTA and DFP

A 100 mM Fe-NTA stock solution was prepared by dissolving 1.64 g NaHCO₃ in 80 mL deionized water, followed by the addition of nitrilotriacetic acid (NTA; 0.256 g) and FeCl₃·6H₂O (0.27 g) under acidic conditions with constant stirring. The pH was adjusted to 6.5 with 10 N NaOH, the volume brought to 100 mL, and the solution filter-sterilized (0.22 µm). DFP was prepared at 100 mM in L-15 medium by gentle stirring overnight and sterilized by filtration (0.22 µm).

### Statistical analyses

Statistical analyses were performed using one-way ANOVA or unpaired t-tests, with significance set at *P* < 0.05. Analyses and graph generation were conducted in GraphPad Prism v10.5 (GraphPad Software, Inc.). To evaluate the effect of infection time on T4SS gene expression, a two-way ANOVA was performed considering dpi and gene as fixed factors, including their interaction (dpi × gene). When significant effects were detected, Tukey’s post hoc tests were applied for pairwise comparisons between dpi levels within each gene. These analyses were performed in R v4.3.2 using the tidyverse and rstatix packages.

## RESULTS

### Standardized infection protocol for dual RNA-seq experiments

**Fig 1:**
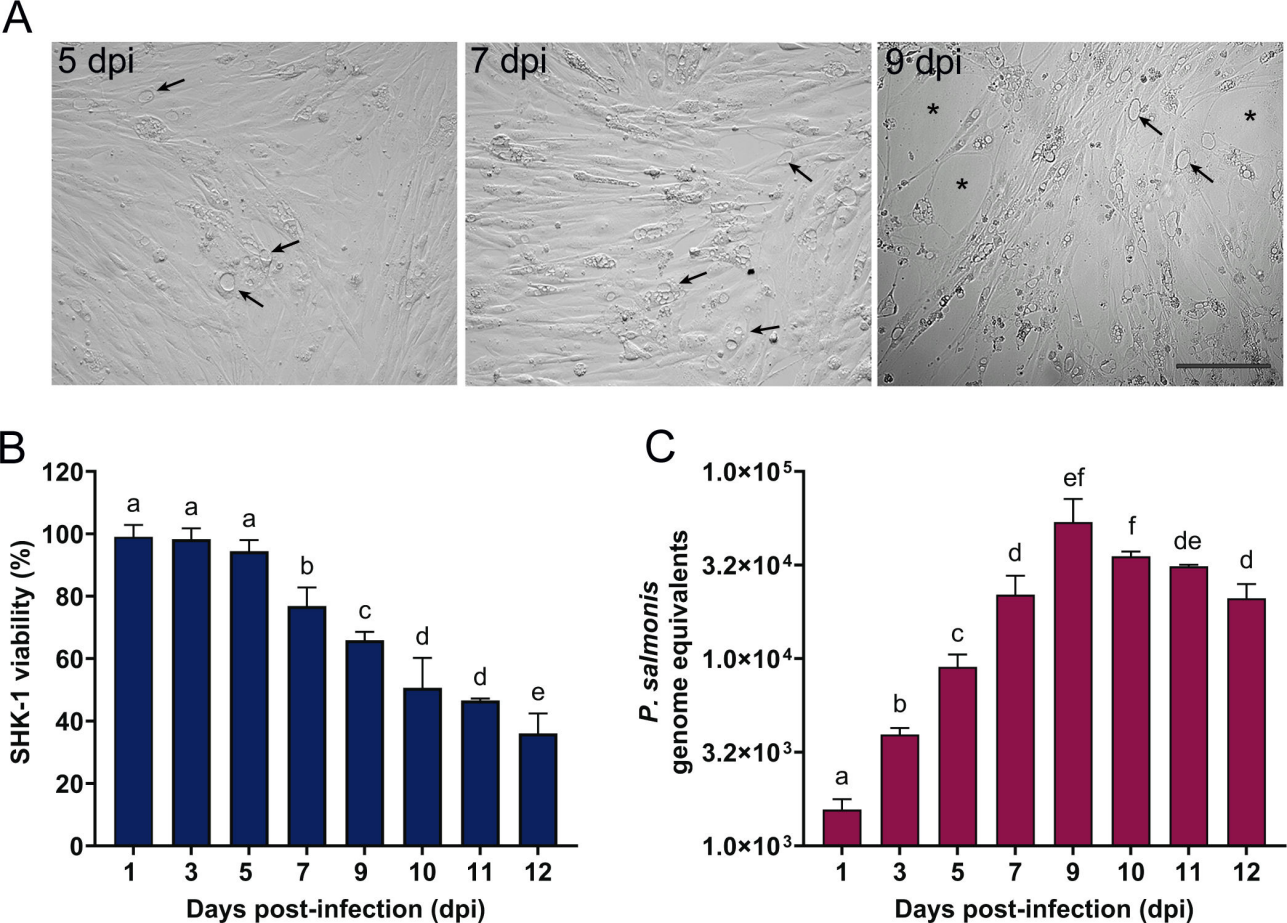
*In vitro* infection of SHK-1 cells. (**A**) Representative bright-field microscopy images of *P. salmonis*-infected cells (arrows) at 5, 7, and 9 dpi. Scale bar 100 µm. (**B**) Cell viability (%) was analyzed with the alamarBlue reagent on different days post-infection. Data reflect means ± SD (*N* = 12 biological replicates). (**C**) Genome equivalents of *P. salmonis* during infection of SHK-1 cells. For each sample, an equal amount of total gDNA was analyzed by quantitative qPCR with a TaqMan probe designed for the *recF* gene. Data reflect means ± SD (*N* = 3 biological replicates and three technical replicates). Different letters indicate statistically significant differences between groups (*P* < 0.05), as determined by one-way ANOVA followed by Tukey’s post hoc test. Groups sharing the same letter are not significantly different.

To characterize the intracellular infection cycle of *P. salmonis* in SHK-1 cells, infection progression was monitored by bright-field microscopy, cell viability assays, and quantification of bacterial genome equivalents. As previously described (5), productive infection was defined by the presence of bacteria within a membrane-bound vacuolar compartment in the host cell cytoplasm, referred to as the *P. salmonis*-containing vacuole (PCV; Fig. 1A, arrows). PCVs were detectable from 5 dpi and increased in frequency over time, being observed in approximately 30%–40% of cells by 7 dpi. At later stages of infection (9 dpi), cytopathic effects became evident, including disruption of the cell monolayer (Fig. 1A, asterisks). Consistent with these observations, host cell viability remained high up to 7 dpi (approximately 80% relative to uninfected controls, Fig. 1B) but declined significantly thereafter, reaching 65% and 30% at 9 and 12 dpi, respectively.

Quantification of bacterial genome equivalents revealed a progressive increase in intracellular *P. salmonis* between 1 and 9 dpi, with maximal bacterial loads detected at 9 dpi (Fig. 1C). This increase was followed by a decline that paralleled the loss of host cell viability at later time points. Based on the combination of high bacterial burden, preserved host cell viability, and widespread PCV formation, 7 dpi was selected for transcriptomic analyses. This time point represents the intracellular replicative phase of *P. salmonis* and provides an appropriate basis for comparison with exponentially growing bacteria cultured in Nutrient *Piscirickettsia* Broth (NBP).

### Dual RNA-seq analysis of *P. salmonis*-infected SHK-1 cells

To characterize host and bacterial transcriptional responses during intracellular infection, dual RNA-seq was performed on *P. salmonis*-infected SHK-1 cells at 7 dpi, corresponding to the intracellular replicative phase. Transcriptomes of intracellular bacteria and infected host cells were compared with those of extracellular *P. salmonis* grown in NPB during exponential phase and uninfected SHK-1 cells, respectively (Fig. S1).

Illumina sequencing generated approximately 342 million reads (M reads) from triplicate libraries of infected SHK-1 cells, with a mean of 114 ± 12 M reads per sample. Of these, ~219 M reads mapped uniquely to the *Salmo salar* genome, corresponding to an average of 73 ± 7 M reads per replicate and representing 69%, 67%, and 59% of uniquely mapped reads across the three libraries. From the remaining reads, more than 7.3 M reads mapped uniquely to the *P. salmonis* CGR02 genome, with an average of 2.5 ± 0.9 M reads per replicate, accounting for 5%, 6%, and 7% of mapped reads, respectively. For cDNA libraries derived from uninfected SHK-1 cells, approximately 83 M reads mapped uniquely to the *S. salar* genome, with a mean of 28 ± 5 M reads per sample (62%, 59%, and 62% of total reads per library). In cDNA libraries generated from exponentially growing *P. salmonis* cultures, more than 32 M reads mapped uniquely to the bacterial genome, averaging 11 ± 1 M reads per sample, with ~95% of reads mapping uniquely to the CGR02 genome in all cases (Table S1). Non-metric multidimensional scaling (NMDS) revealed clear separation between intracellular and extracellular *P. salmonis* transcriptomes, as well as between infected and uninfected host cells, with high reproducibility among biological replicates (mean correlation > 0.98) (Fig. S2A and B). Differential expression measured by RNA-seq was validated by RT-qPCR for selected bacterial and host genes, showing strong concordance between both approaches (*R*² = 0.83; Fig. S3A and B, the genes tested by RT-qPCR are highlighted in black in Tables S3 and S4, list of primers in Table S2).

### Intracellular transcriptional response of *P. salmonis*

**Fig 2:**
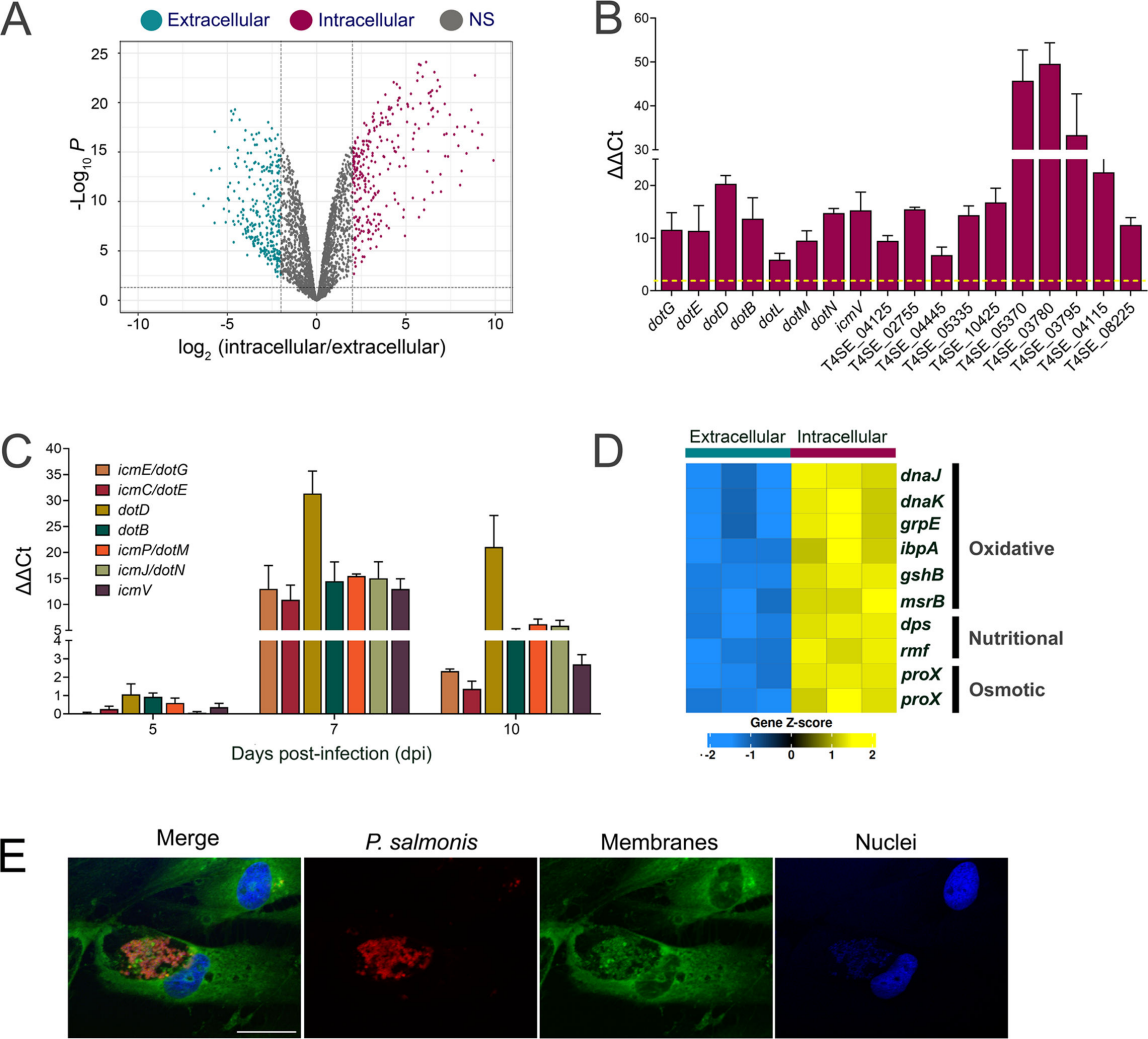
Transcriptomic response of *P. salmonis* during intracellular growth. (**A**) Volcano plot from DESeq2 analysis of extracellular and intracellular *P. salmonis* RNA pools. The red dots represent significantly upregulated genes, the green dots represent significantly downregulated genes (|log_2_FC| > 2, adj. *P* value < 0.05) in the intracellular *P. salmonis*. Gray dots: no significant change of expression. (**B**) RT-qPCR verification of differential expression of genes encoding components of the T4BSS and predicted effectors of the T4BSS (T4SE_locus number). The fold change in gene expression was calculated using the ΔΔCt method. The dotted line indicates a two-fold change in expression. The mean ± SD from three independent experiments is shown. (**C**) Differential expression of *P. salmonis* genes encoding components of the T4BSS was analyzed by RT-qPCRs at 5, 7, and 10 dpi. Fold change in gene expression was calculated using the ΔΔCt method. Two-way ANOVA showed significant differences with regard to dpi (*F* = 128.5, *P* = <2 × 10^−18^), ΔΔCt value (*F* = 15.4, *P* = 3 × 10^−9^) and interaction effect (*F* = 3.6, *P* = 9 × 10^−4^). (**D**) Heatmap illustrating the expression values (z-score) of genes associated with the response of bacteria to different stresses. Complete description of genes in Table S3. (**E**) Representative image of indirect immunofluorescence using a specific antibody against *P. salmonis* (red). SHK-1 cells were stained with the membrane stain FM1-43Fx (green) and DAPI to detect DNA (blue). Bar = 25 µm.

Comparison of intracellular and extracellular *P. salmonis* transcriptomes identified 561 differentially expressed genes (|log₂FC| > 2, adj. *P* < 0.05), of which 270 were upregulated, and 291 were downregulated during intracellular growth (Fig. 2A). Genes upregulated intracellularly were significantly enriched in functions related to host interaction and stress adaptation. Among the most strongly induced genes were those encoding components of the Dot/Icm type IVB secretion system (T4BSS) (Table S3). Bioinformatic analyses identified 28 genes encoding putative T4BSS effectors (T4SEs) that were significantly upregulated during intracellular infection (log₂FC > 2, adj. *P* < 0.05). These predictions were made using consensus predictions from different computational tools. RT-qPCR analysis confirmed the intracellular induction of T4BSS structural genes and predicted effectors (Fig. 2B; Table S3, list of primers in Table S2). To assess the temporal dynamics of T4BSS expression, transcript levels were measured by RT-qPCR at 5, 7, and 10 dpi (Fig. 2C). Two-way ANOVA analysis indicated statistically significant differences in the expression of all T4BSS components among 5, 7, and 10 dpi. A peak in expression was observed at 7 dpi for all the tested genes, with particularly marked differences detected for *dotD*, *dotM*, *dotN,* and *dotG*. At 10 dpi, with the exception of *dotD*, the expression of all the genes decreased significantly. These results suggest a time-restricted induction of the T4BSS during intracellular infection.

In addition to secretion-related genes, intracellular *P. salmonis* exhibited increased expression of genes associated with stress responses (Fig. 2D; Table S3). These included genes involved in oxidative stress management (*dnaK*, *dnaJ*, *grpE*, *ibpA*, *gshB*, and *msrB*), nutrient limitation (*dps*, encoding the DNA-binding protein from starved cells, and *rmf*, encoding the ribosome modulation factor), and osmoprotection (*proX*). In addition, approximately half of the top 20% most highly expressed genes encoded hypothetical proteins or proteins with limited functional annotation, suggesting the involvement of uncharacterized factors during intracellular adaptation (Table S3). Together, the transcriptional profile of intracellular *P. salmonis* is characterized by coordinated induction of secretion system components, predicted effectors, and genes associated with adaptation to stressful intracellular conditions. These changes occur during active bacterial replication within large *P. salmonis*-containing vacuoles that expand throughout the host cell cytoplasm (Fig. 2E), linking the observed gene expression program to the defined intracellular niche in which replication takes place.

### Host transcriptional and functional activation of lysosomal pathways during *P. salmonis* infection

**Fig 3:**
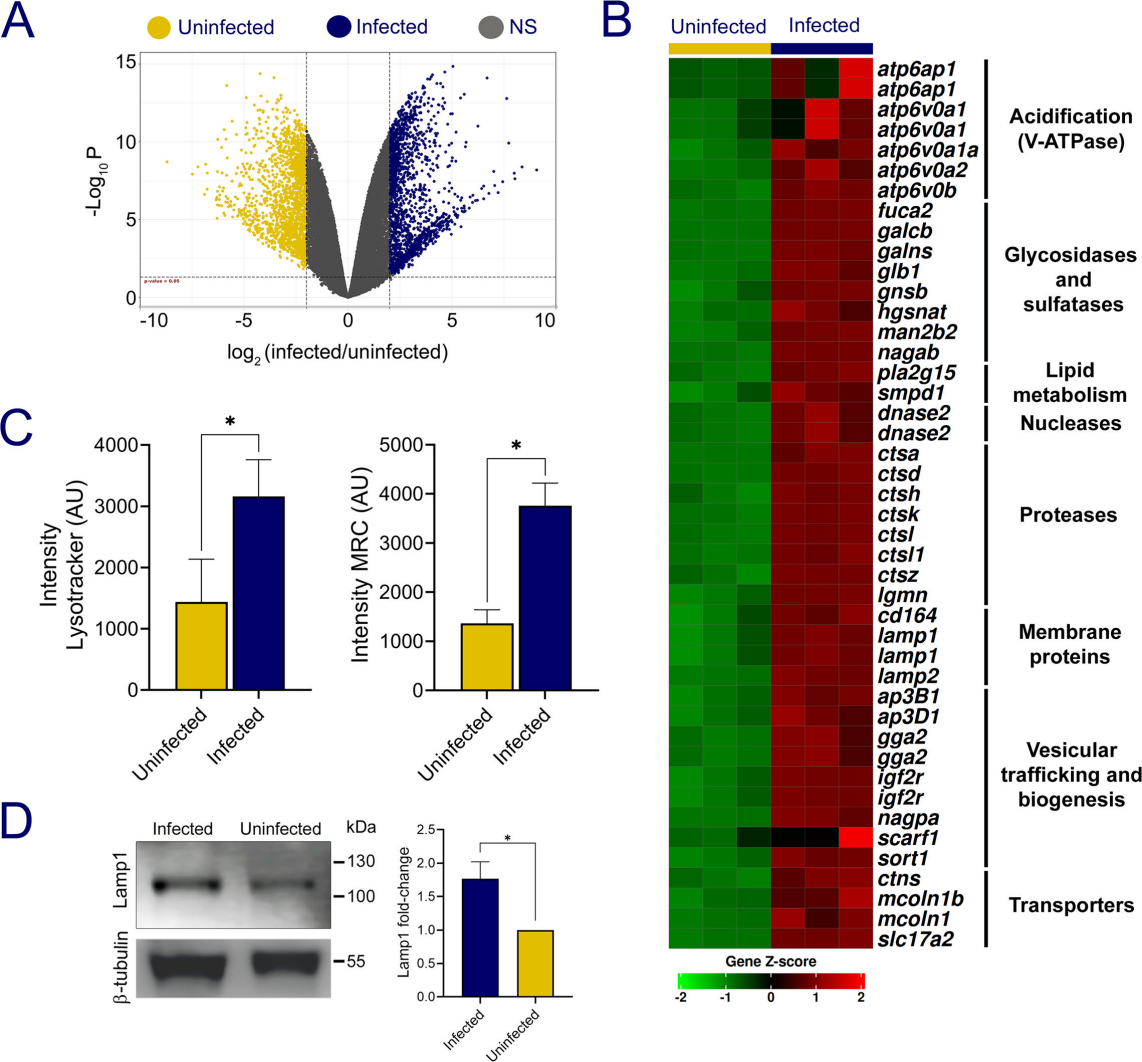
The host response to *P. salmonis* infection. (**A**) Volcano plots of DESeq2 results based on RNAseq analysis of uninfected and infected SHK-1 cells. The blue dots represent significantly upregulated genes, the yellow dots represent significantly downregulated genes (|log_2_FC| > 2, adj. *P* value < 0.05) in *P. salmonis*-infected SHK-1 cells. Gray dots: no significant change of expression. (**B**) Heatmap of expression values (z-score) for 44 genes associated with KEGG pathway sasa04142, and classified into main functional categories. Complete description of genes in Tables S4 and S5. (**C**) SHK-1 cells that were infected (blue box) or uninfected (yellow box) with *P. salmonis* were labeled with LysoTracker Red (left graph) or with Magic Red Cathepsin B (right graph) at 7 dpi. The graphs show the mean total fluorescence intensity of at least 90 randomly selected cells. Statistical significance was assessed using t-test, * denotes *P* < 0.05. (**D**) The levels of Lamp-1 protein in *P. salmonis*-infected and uninfected SHK-1 cell lysates were evaluated using immunoblotting with an anti-Lamp-1 antibody. β-Tubulin was used as a loading control. A representative image of the western blot result is shown in the left panel, and the graph displays the mean and standard deviation of Lamp-1 band intensity, normalized to β-Tubulin, from three biological replicates. Statistical significance was assessed using t-test, * denotes *P* < 0.05.

Differential gene expression analysis of *P. salmonis*–infected SHK-1 cells identified a total of 1,692 upregulated and 1,790 downregulated host genes (|log₂FC| > 2, adj. *P* value< 0.05) compared with uninfected cells (Fig. 3A, blue dots). Genes upregulated upon infection were significantly enriched in functional categories related to innate and adaptive immune responses, extracellular matrix remodeling, transmembrane transport, actin cytoskeleton organization, and apoptosis (Table S4). KEGG pathway enrichment analysis revealed overrepresentation of pathways associated with cell–extracellular matrix interactions (sasa04512), immune signaling (sasa04625, C-type lectin receptor signaling), and phagocytosis (sasa04145) (Table S5). Infection also induced a strong transcriptional signature related to lysosome biogenesis and activity (sasa04142: Lysosome). This group comprised 42 differentially expressed genes associated with lysosomal pathways, including those encoding mannose-6-phosphate receptors (M6PR), vacuolar ATPase (V-ATPase) subunits, hydrolases such as cathepsins, and membrane proteins, including Lamp-1 and Lamp-2 (Fig. 3B; Table S5, tab KEGG_Lysosome). Twenty-nine of these genes have orthologs that were previously identified in metazoans as part of the gene network activated by the transcription factor EB (TFEB target gene in Table S5), a master regulator of genes encoding proteins involved in lysosome biogenesis and function (44). Differential expression of 11 lysosome-associated genes was validated by RT-qPCR (Fig. S3B).

To determine whether these transcriptional changes were reflected at the functional level, lysosomal properties were examined in infected and uninfected SHK-1 cells by staining with the acidotropic dye LysoTracker Red, which accumulates in organelles with acidic pH (45). The results showed that at 7 dpi, infected cells have, on average, significantly more LysoTracker fluorescence than uninfected cells (*P* < 0.05, Fig. 3C, left graph). Representative images of infected and uninfected cells after LysoTracker staining are shown in Fig. S4. In parallel, lysosomal proteolytic activity was assessed using the Magic Red Cathepsin B (MRC) probe, a cell-permeable fluorogenic substrate that emits fluorescence upon cleavage by the lysosomal cysteine protease cathepsin B. Infected cells displayed significantly higher fluorescence compared with uninfected controls, demonstrating increased cathepsin B activity (Fig. 3C, right panel; Fig. S4).

Consistent with these observations, immunoblot analysis showed elevated levels of lysosome-associated membrane protein 1 (Lamp-1) in infected cells relative to uninfected controls (Fig. 3D), confirming an increase in lysosomal content during infection. Together, transcriptomic, imaging, and biochemical analyses indicate that *P. salmonis* infection elicits a robust lysosomal response in SHK-1 cells, characterized by enhanced lysosome biogenesis, acidification, and proteolytic activity.

### *P. salmonis* replicates within Lamp-1–positive, acidic vacuoles and retains infectivity after intracellular passage

**Fig 4:**
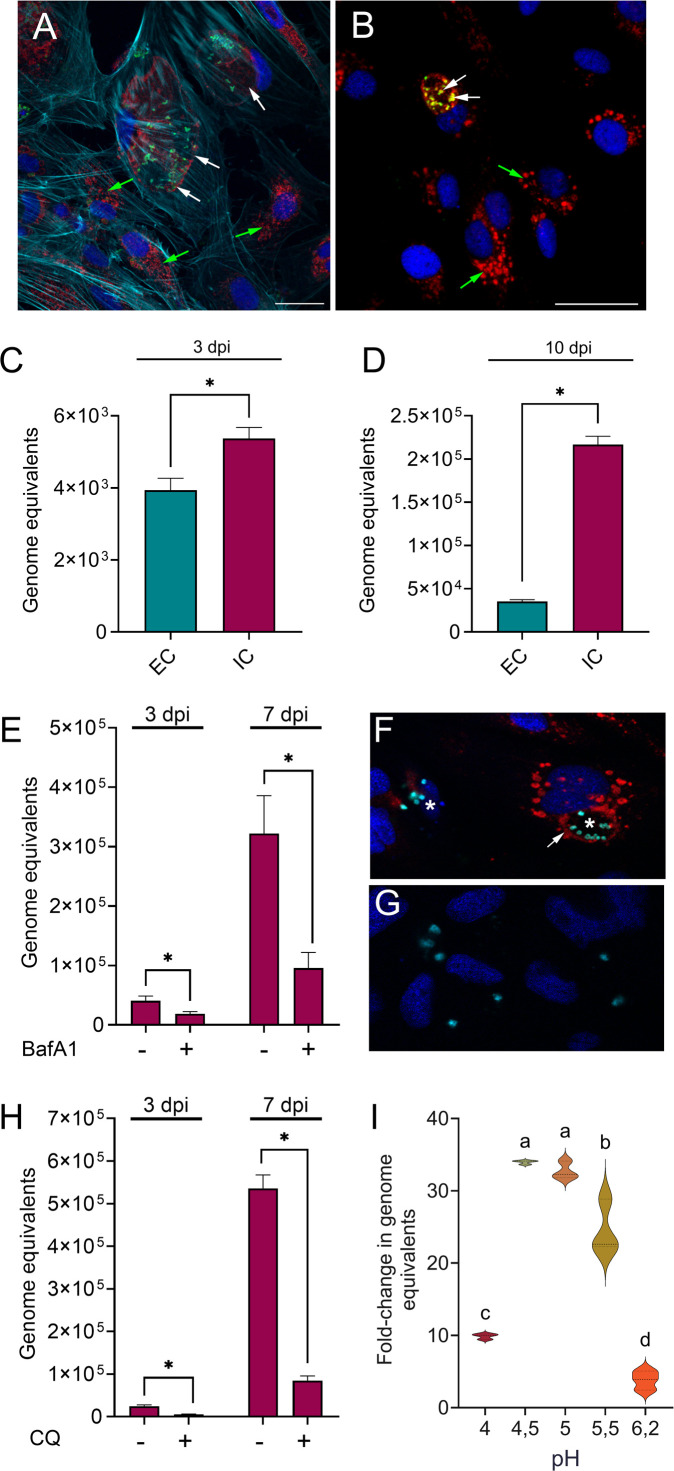
Intracellular *P. salmonis* survives the harsh environment of the vacuole. (**A**) Representative immunofluorescence image of *P. salmonis*-infected SHK-1 cells at 7 dpi stained with antibodies against *P. salmonis* (green) and Lamp-1 (red), Alexa Fluor 647 Phalloidin (cyan), and DAPI (blue). White arrows indicate Lamp-1 localization at the PCVs, and green arrows point to uninfected cells. Scale bar = 50 µm. (**B**) Representative immunofluorescence image of *P. salmonis*-infected SHK-1 cells at 7 dpi stained with antibodies against *P. salmonis* (green), with the acidotropic dye LysoTracker Red DND-99 (red), and DAPI (blue). White arrows indicate co-localization between *P. salmonis* and LysoTracker at the PCV (the co-localization is visualized as dots in yellow color), green arrows point to uninfected cells. Scale bar = 50 µm. (**C-D**) SHK-1 cells were infected with extracellular *P. salmonis* (EC, grown in broth to the stationary phase) or with intracellular *P. salmonis* recovered at 7 dpi (IC). Genome equivalents of intracellular *P. salmonis* were evaluated at 3 and 10 dpi. Data reflect means ± SD (*N* = 3 biological replicates per condition). Statistical significance was assessed using t-test, * denotes *P* < 0.05. (**E**) Genome equivalents of intracellular *P. salmonis* at 3 and 7 dpi with or without a 24 h pre-incubation with BafA1. Data reflect means ± SD (*N* = 3 biological replicates). Statistical significance was assessed using t-test, * denotes *P* < 0.05. (**F and G**) Representative immunofluorescence images of SHK-1 cells infected with *P. salmonis* for 7 days in the absence (**F**) or presence (**G**) of 100 µM BafA1. Antibodies against *P. salmonis* (cyan), LysoTracker Red DND-99 (red), and DAPI (blue) are shown. In panel (**F**), vacuoles with clusters of replicating bacteria can be observed (asterisks), which are also labeled with LysoTracker Red DND-99 (arrow). (**H**) Genome equivalents of intracellular *P. salmonis* at 3 and 7 dpi, with or without a 24 h pre-incubation with CQ. Data reflect means ± SD (*N* = 3 biological replicates). Statistical significance was assessed using t-test, * denotes *P* < 0.05. (**I**) Genome equivalents were calculated at the start of the culture and again 50 h later. Different letters indicate statistically significant differences between groups (*P* < 0.05), as determined by one-way ANOVA followed by Tukey’s post hoc test. Groups sharing the same letter are not significantly different.

To further characterize the intracellular niche supporting *P. salmonis* replication, we examined the association of Lamp-1 with PCVs at 7 dpi, a time point at which Lamp-1 expression is upregulated in infected cells. Immunofluorescence analysis revealed strong Lamp-1 localization at the PCVs (representative image in Fig. 4A, white arrows), whereas in uninfected cells, Lamp-1 displayed a typical perinuclear punctate distribution (Fig. 4A, green arrows). Quantitative analysis showed that more than 90% of PCVs were Lamp-1–positive at 7 dpi. Furthermore, when SHK-1 cells infected with *P. salmonis* were incubated at 7 dpi with LysoTracker Red DND99 and stained with an anti-*P*. *salmonis* antibody, confocal fluorescence imaging revealed that 56% of the PCVs were LysoTracker positive (representative image in Fig. 4B). Given that these Lamp-1–positive, acidified vacuoles are generally associated with antimicrobial activity, we next assessed bacterial viability at this stage of infection. Using the most-probable number (MPN) method, we recovered approximately 4.2 × 10⁷ viable bacteria/mL from infected cells at 7 dpi, confirming that *P. salmonis* remains viable and replicative at this stage.

We then examined whether passage through the intracellular environment influences bacterial infectivity in subsequent infection cycles. Intracellular *P. salmonis* recovered at 7 dpi (IC) or extracellular bacteria grown to the stationary phase in broth (EC) were used to infect naïve SHK-1 cells. Compared with EC bacteria, IC bacteria displayed significantly enhanced intracellular replication, with a 1.4-fold increase at 3 dpi and a 6.1-fold increase at 10 dpi (Fig. 4C and D). These results indicate that intracellular passage enhances the capacity of *P. salmonis* to replicate during subsequent infections.

To evaluate the contribution of vacuolar acidification to intracellular replication of *P. salmonis*, SHK-1 cells were treated with bafilomycin A1 (BafA1), an inhibitor of vacuolar ATPases (46), or chloroquine (CQ), a lysosomotropic weak base (47, 48). Control experiments confirmed that neither compound affected bacterial viability, host cell viability, or phagocytic capacity at the concentrations used (Fig. S5). Inhibition of vacuolar acidification by BafA1 resulted in a significant reduction in intracellular bacterial genome equivalents at both 3 and 7 dpi (Fig. 4E). Consistently, LysoTracker-positive vacuoles containing bacterial clusters were readily observed in untreated cells (Fig. 4F, asterisks) but were absent in BafA1-treated cells (Fig. 4G). Similarly, CQ treatment significantly reduced intracellular replication of *P. salmonis*, resulting in 4.9-fold and 6.4-fold decreases in bacterial genome equivalents at 3 and 7 dpi, respectively (Fig. 4H).

To further assess the capacity of *P. salmonis* to survive and replicate under acidic conditions, bacteria were cultured in defined CMMAB medium (12) adjusted to pH values ranging from 4.0 to 6.2. Maximal increases in genome equivalents after 50 h were observed at pH 4.5 and 5.0 (34-fold and 32-fold, respectively), whereas growth was reduced at pH 4.0 and progressively decreased at higher pH values (22.6 and 5.0 at pH 5.5 and 6.2, respectively) (Fig. 4I). These results indicate that *P. salmonis* can tolerate and replicate under mildly acidic conditions, which is compatible with its intracellular replication within acidic vacuolar compartments.

### SHK-1 cell iron content affects intracellular replication of *P. salmonis*

**Fig 5:**
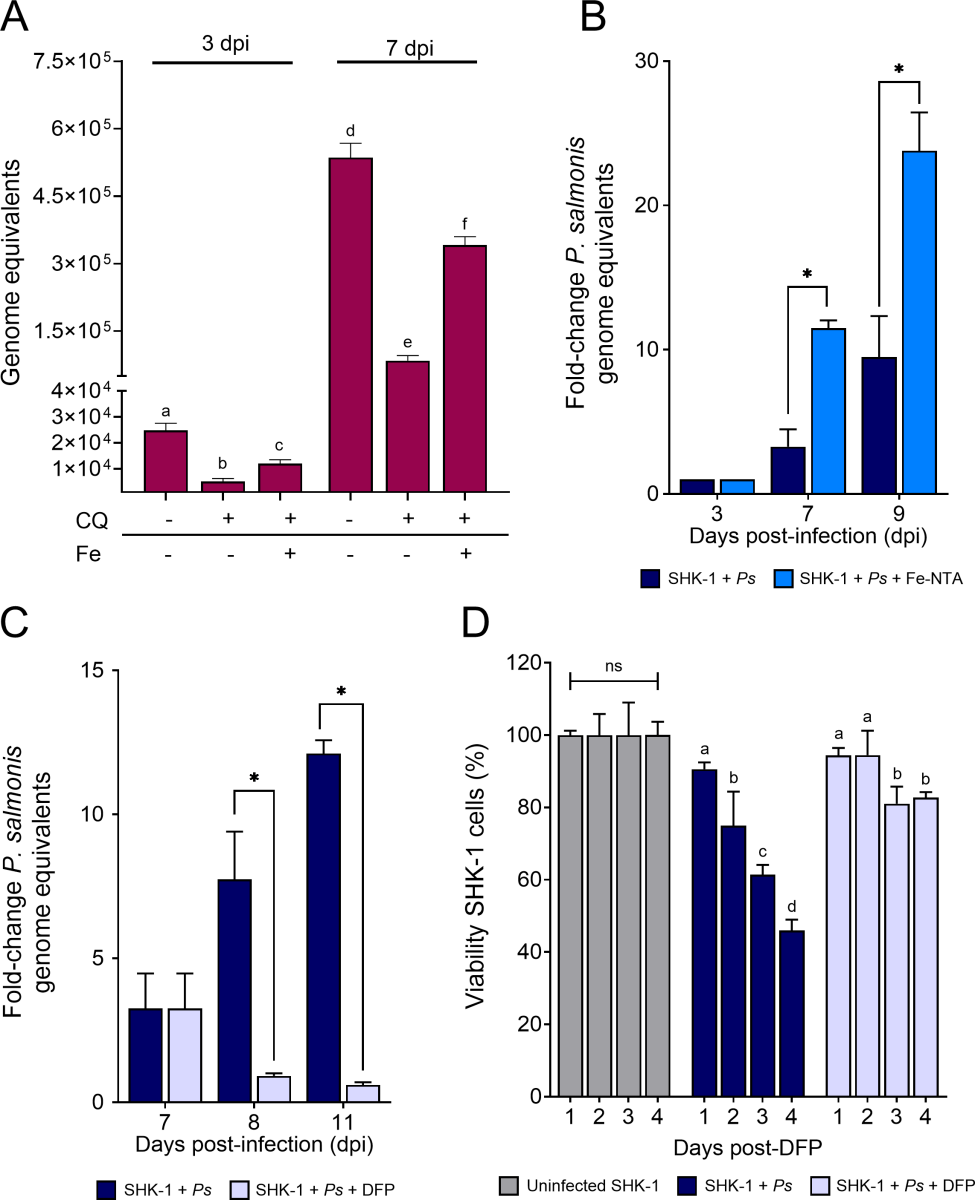
Host cell iron availability influences intracellular *P. salmonis* replication. (**A**) Genome equivalents of *P. salmonis* at 3 and 7 dpi. SHK-1 cells were pre-incubated with chloroquine (CQ) and subsequently infected with *P. salmonis* in the absence or presence of 25 µM Fe–NTA. Data reflect means ± SD (*N* = 3 biological replicates). Statistical significance was assessed by one-way ANOVA; different letters indicate significant differences (*P* < 0.05). (**B**) SHK-1 cells were supplemented or not with 25 µM Fe–NTA for four days prior to infection with *P. salmonis*. Fold-change values were calculated by normalizing genome equivalents at 3, 7, and 9 dpi to those at 3 dpi. Data correspond to the mean ± SD of three biological replicates. Statistical significance was assessed by an unpaired t-test; * denotes *P* < 0.05. (**C**) SHK-1 cells were infected with *P. salmonis*, and at 7 dpi were treated or not with 100 µM DFP. Fold-change values were calculated by normalizing genome equivalents at 8 and 11 dpi to those at 7 dpi. Data correspond to the mean ± SD of three biological replicates. Statistical significance was assessed by an unpaired t-test; * denotes *P* < 0.05. (**D**) SHK-1 cells were infected with *P. salmonis* or heat-inactivated bacteria. At 7 dpi, cells were exposed or not to 100 µM DFP for 4 days, and cell viability was assessed at the indicated time points following DFP treatment. Data represent mean ± SD (*N* = 6 biological replicates). Statistical significance was evaluated by one-way ANOVA followed by Tukey’s post hoc test; groups with different letters are significantly different (*P* < 0.05), whereas groups sharing at least one letter are not significantly different; ns, not significant.

Because inhibition of vacuolar acidification by BafA1 or CQ can impair transferrin-mediated iron release in host cells (49–51), we examined whether altered iron availability contributes to the reduced replication of *P. salmonis* under these conditions. SHK-1 cells were treated with CQ prior to infection and supplemented or not with 25 µM Fe-NTA, a soluble iron source whose availability is independent of acidic pH. At both 3 and 7 dpi, Fe-NTA supplementation partially restored genome equivalents in CQ-treated cells (Fig. 5A), indicating that reduced iron availability partially contributes to the inhibitory effect of CQ.

To directly assess the impact of host cell iron status on *P. salmonis* replication. SHK-1 cells were rendered moderately iron deficient by treatment with the iron chelator deferiprone (DFP; 100 µM) or iron loaded by supplementation with Fe-NTA (25 µM). Neither treatment affected host cell viability under the conditions used (Fig. S6A and B). In agreement with altered intracellular iron levels, the expression of the transferrin receptor (TFR), which is regulated by iron availability in eukaryotic cells (52), increased following DFP treatment and decreased upon Fe-NTA supplementation (Fig. S6C).

Iron loading of SHK-1 cells prior to infection resulted in a significant increase in *P. salmonis* genome equivalents at 7 and 9 dpi relative to 3 dpi (Fig. 5B), indicating enhanced intracellular replication under elevated iron conditions. Conversely, chelation of intracellular iron during the replicative phase significantly restricted bacterial growth. When infected cells were treated with DFP at 7 dpi and monitored for 4 additional days, bacterial genome equivalents decreased by 7.7-fold at 8 dpi and 12-fold at 11 dpi relative to 7 dpi (Fig. 5C). DFP treatment was also associated with significantly improved host cell viability compared with untreated infected controls (Fig. 5D), suggesting that iron chelation reduced infection progression while allowing recovery of SHK-1 cells.

In line with these functional observations, RNA-seq analysis revealed upregulation of several *P. salmonis* genes involved in iron acquisition and utilization during intracellular infection (Table S3; Fig. S7). These included components of ferrous and ferric iron transport systems (*feo*, *fep*), the bacterioferritin-associated ferredoxin (*bfd*), genes encoding putative siderophore biosynthesis enzymes (*pvsA/D*), and the TonB–ExbB–ExbD energy transduction complex. Together, these data indicate that intracellular replication of *P. salmonis* is associated with host cell iron availability and induction of bacterial iron-acquisition pathways.

## DISCUSSION

In this study, we used a dual RNA-seq strategy, combined with biochemical and cell biological analyses to define the intracellular niche of *P. salmonis* and characterize the coordinated host and bacterial responses that accompany intracellular replication. Our results provide a comprehensive view of how *P. salmonis* survives and replicates within macrophage-like SHK-1 cells despite activation of host antimicrobial pathways.

A central feature of the host response to *P. salmonis* infection was the robust transcriptional activation of lysosome-associated pathways. Infected SHK-1 cells upregulated genes involved in lysosome biogenesis, acidification, and proteolytic activity. This lysosomal transcriptional program aligns with activation of the TFEB-regulated CLEAR network (44). TFEB is a master regulator of lysosome biogenesis (53, 54) whose activation has been shown to exert divergent effects on intracellular pathogens, promoting vacuolar expansion and replication in *Coxiella burnetii* (55) while contributing to bacterial restriction in *Salmonella enterica* serovar Typhimurium (56). In the case of *P. salmonis* infection, our results suggest that induction of lysosomal programs may occur alongside sustained intracellular bacterial replication.

Consistent with the transcriptomic data, functional assays demonstrated increased lysosomal acidification and cathepsin activity in infected cells. Importantly, *P. salmonis* localized predominantly within Lamp-1–positive vacuoles that retained acidic properties. As Lamp-1 and Lamp-2 proteins are critical for the transition from early to late phagosomes (57), these observations suggest that the *P. salmonis*-containing vacuole matures along the endocytic pathway and acquires classical late endosomal and lysosomal features. Notably, *P. salmonis* induces extensive vacuolar expansion, with large vacuoles occupying much of the host cell cytoplasm. This marked expansion suggests active remodeling of the intracellular compartment, while the persistence of acidic and lysosomal characteristics distinguishes the *P. salmonis* vacuole from those of pathogens that neutralize or evade lysosomal maturation (58, 59).

Residence within an acidic vacuole has been documented for several intracellular pathogens at defined stages of infection. In the case of *Legionella pneumophila*, vacuolar acidification becomes important during the late stages of infection, which coincides with the acquisition of Lamp-1 and other lysosomal markers. Meanwhile, the pharmacological inhibition of acidification restricts replication (60). Similarly, *Brucella abortus*-containing vacuoles travel through the endocytic pathway and temporarily fuse with lysosomes. Acidification is required at this stage for the survival and replication of the bacteria, as well as for subsequent maturation of the replicative niche and activation of the type IV secretion system (61, 62). *Coxiella burnetii* represents the most extreme example, as it depends on a persistently acidic vacuole for metabolic activation and Dot/Icm-mediated effector translocation (63–65). In this context, our results show that inhibition of vacuolar acidification by bafilomycin A1 or chloroquine significantly restricts intracellular replication of *P. salmonis*, indicating that acidic conditions are functionally important for its intracellular growth. Whether vacuolar acidification is also required for optimal activity of the *P. salmonis* type IVB secretion system and its effector proteins remains to be determined. Interestingly, early studies reported that transient acidification of cell-free medium induces the expression of T4BSS components in *P. salmonis* (10), suggesting a potential link between acidic cues and secretion system regulation.

Taken together, these observations are notable, given that this pathogen grows optimally in nutrient broth at near-neutral pH (6.2–7.0) (12, 66, 67). Nonetheless, our data indicate that *P. salmonis* can replicate under mildly acidic conditions *in vitro*, which is compatible with the acidic characteristics of the PCV. Exposure to the intracellular environment may activate cross-protection responses that enhance bacterial resilience to multiple adverse conditions, including low pH, oxidative stress, and nutrient limitation (68). Such stress-induced physiological plasticity could contribute to the ability of *P. salmonis* to persist and replicate within acidic intracellular compartments.

At the bacterial level, intracellular replication was associated with marked induction of genes encoding the Dot/Icm type IVB secretion system (T4BSS). T4BSSs are conserved virulence determinants among a phylogenetically related group of intracellular Gammaproteobacteria (69), including the order Legionellales and the families Francisellaceae and Piscirickettsiaceae, in which the T4BSS can be traced to a common ancestor (69–73). Although the secretion machinery itself is highly conserved, the repertoire of T4BSS effectors (T4SEs) is typically more variable and exhibits limited conservation across species. During intracellular growth of *P. salmonis,* we identified 28 upregulated genes predicted as putative T4SEs. Most (23/28) encode hypothetical proteins without assigned function, whereas the remaining candidates include proteins with predicted kinase domains or tetratricopeptide repeats. Four putative kinases showed sequence homology to known secreted effectors in the Virulence Factor Database, and secreted kinases in other intracellular pathogens have been implicated in cytoskeletal remodeling and modulation of host immune signaling pathways (74). T4BSS gene expression peaked around 7 dpi, coinciding with the intracellular replicative phase defined in our experimental model. Thus, the temporal pattern of expression suggests that the T4BSS contributes primarily to adaptation and maintenance of the established intracellular niche rather than to early invasion events.

In addition to secretion systems, intracellular *P. salmonis* exhibited induction of a defined set of genes associated with stress resistance and intracellular survival. These included classical molecular chaperones and co-chaperones (*dnaK*, *dnaJ*, *grpE*, and *ibpA*), which mediate protein folding and protect against denaturation under acidic and heat stress conditions (75, 76). DnaK has been implicated in the survival of pathogenic bacteria within macrophages (77), and its induction in response to low pH has been documented in *Brucella melitensis* (78). Additional upregulated genes involved in oxidative stress resistance and redox homeostasis included *gshB*, which catalyzes the final step of glutathione biosynthesis, and *msrB*, responsible for the reduction of Methionine-R-sulfoxide (79–81), supporting exposure to reactive oxygen species within the PCV. Induction of genes associated with nutrient limitation and metabolic downshifts (*dps*, *rmf*) aligns with adaptation to restricted nutrient availability during intracellular growth (82–85). Together, these transcriptional changes indicate that intracellular *P. salmonis* engages coordinated stress-response programs that likely enhance tolerance to the acidic, oxidative, and nutrient-limited conditions of the vacuolar environment, paralleling stress-adaptive strategies described in other intracellular pathogens (68, 86). This stress-associated transcriptional profile may also be linked to the physiological changes observed following intracellular passage. Bacteria recovered from infected cells displayed enhanced replication during secondary infection compared with broth-grown counterparts, suggesting that residence within host cells promotes a differentiated physiological state that favors subsequent intracellular replication. Although the environmental cues and regulatory networks underlying this effect remain to be fully defined, previous studies reported upregulation of stringent response–associated genes during late stages of intracellular growth (5); such regulation may support bacterial survival under prolonged intracellular stress. These observations are compatible with a model in which stress-responsive regulatory pathways contribute not only to immediate intracellular tolerance but also to physiological differentiation across the infectious cycle, thereby enhancing the ability of *P. salmonis* to sustain infection through successive host-cell encounters.

A substantial fraction of the intracellular transcriptional response of *P. salmonis* corresponded to genes lacking functional annotation. Of the 270 genes upregulated during intracellular growth, 96 (30%) encoded proteins of unknown function. Unannotated genes account for approximately one-third of coding sequences in many bacterial genomes (87, 88), and in *P. salmonis,* between 24% and 32% of predicted genes are classified as unknown depending on the strain and annotation data set (89). In the strain analyzed here, 728 of 3,111 genes (24%) lack functional annotation. As proposed for prokaryotic genomes more broadly (90), integrating future transcriptomic data from *P. salmonis* exposed to diverse environmental conditions or genetic perturbations, including co-expression and comparative differential expression analyses, may help provide functional context and enable inference of biological roles for these uncharacterized genes (91).

Our results also indicate that intracellular replication of *P. salmonis* was influenced by host-cell iron availability. Inhibition of vacuolar acidification with bafilomycin A1 or chloroquine significantly reduced bacterial replication, and this reduction was partially alleviated by supplementation with an acidification-independent iron source. This effect is supported by previous reports demonstrating that chloroquine elevates the pH of endocytic and lysosomal compartments, thereby impairing the pH-dependent release of iron from transferrin (92). Beyond limiting iron release, alkalinization of these compartments has been associated with broader metabolic alterations, including shifts in amino acid composition that may further constrain bacterial growth (51, 93). Although the precise mechanisms by which bafilomycin A1 and chloroquine restrict *P. salmonis* replication remain to be fully defined, the observed enhancement of replication by iron supplementation and its reduction by iron chelation support a functional role for iron availability during intracellular growth. RNA-seq analysis further revealed the induction of several iron-acquisition systems during intracellular replication, including ferrous and ferric transport components and the *bfd* gene, commonly upregulated under iron-limiting conditions (94, 95). Moreover, recent application of Mobile-CRISPRi in *P. salmonis* demonstrated that the expression of selected iron-acquisition-related genes can be experimentally modulated, establishing a platform for future functional interrogation of these pathways during infection (96). Collectively, these data indicate that iron acquisition represents a prominent adaptive component of the intracellular transcriptional response.

Vacuolar acidification constitutes an additional defining feature of the intracellular niche associated with efficient bacterial replication in our model. While interference with acidification and experimental manipulation of iron levels both affected intracellular growth, the available data do not resolve whether acidification primarily promotes replication through effects on iron bioavailability or whether these parameters represent partially independent constraints of the vacuolar environment. Clarifying the mechanistic relationship between vacuolar pH and iron homeostasis will require targeted genetic and physiological approaches.

In summary, our findings support a model in which *P. salmonis* replicates within an expanded, Lamp-1–positive vacuole that exhibits characteristics of an acidified late endosomal/lysosomal compartment and nevertheless permits bacterial growth. The available evidence suggests that intracellular replication is associated with tolerance to acidic conditions, induction of stress-response pathways, activation of secretion systems, and adaptation to restricted iron availability. Together, these features define key aspects of the intracellular niche encountered by *P. salmonis* and the corresponding bacterial response. By integrating host and pathogen transcriptional profiles with functional perturbation experiments, this study delineates central elements of the intracellular lifestyle of *P. salmonis* while highlighting mechanistic questions that remain unresolved. The recent development of genetic tools in *P. salmonis*, such as Mobile-CRISPRi, provides an opportunity to directly examine the contribution of specific bacterial genes and pathways identified here to intracellular replication and host–pathogen interactions.

## Data Availability

All sequencing data generated in this study have been deposited in the Sequence Read Archive (SRA) at the National Center for Biotechnology Information (NCBI) under BioProject accession number PRJNA1072728 and in the Gene Expression Omnibus (GEO) database under accession number GSE254974. The R scripts used in this study, along with raw data and figures, are available at https://gitlab.com/chodar/psalmonis.dualrna. This repository also includes Video 1, corresponding to a Z-stack reconstruction that provides a three-dimensional visualization of the intracellular localization of *P. salmonis* within a large vacuolar compartment.

## SUPPLEMENTAL MATERIAL

Supplemental figures msystems.00223-26-s0001.pdf Fig. S1-S7.

Table S1 msystems.00223-26-s0002.xlsx Mapping summary.

Table S2 msystems.00223-26-s0003.pdf Sequences of primers used for RT-qPCR.

Table S3 msystems.00223-26-s0004.xlsx Genes differentially expressed between intracellular and extracellular *P. salmonis*.

Table S4 msystems.00223-26-s0005.xlsx Genes differentially expressed between *P. salmonis*-infected and uninfected SHK-1 cells and GO annotation.

Table S5 msystems.00223-26-s0006.xlsx KEGG pathway enrichment analysis of genes differentially expressed in *P. salmonis*-infected SHK-1 cells.

ASM does not own the copyrights to Supplemental Material that may be linked to, or accessed through, an article. The authors have granted ASM a non-exclusive, world-wide license to publish the Supplemental Material files. Please contact the corresponding author directly for reuse.
