# Supplementary material for: Dual transcriptional analysis provides insights into the replicative niche of P. salmonis and the host response during infection
Source: mSystems. 2026 Apr 20;11(5):e00223-26. doi: 10.1128/msystems.00223-26 (PMC13185642; doi:10.1128/msystems.00223-26)
Supplement: Supplemental figures — Fig. S1-S7. [file msystems.00223-26-s0001.pdf]

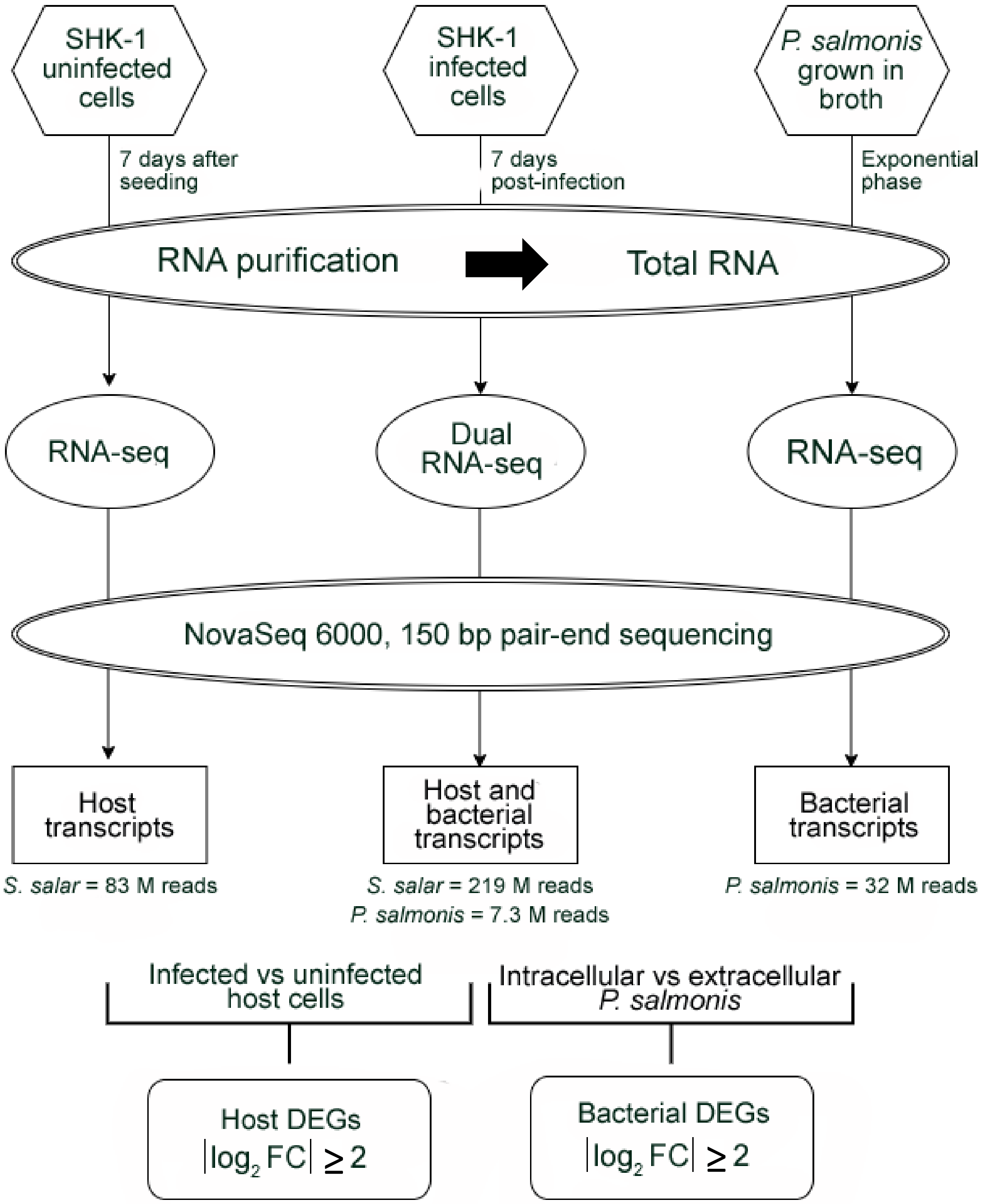

Fig S1. Experimental design for RNA-seq and dual RNA-seq analyses. Analysis were carried out for uninfected SHK-1 cells collected 7 days after seeding, SHK-1 cells infected with *P. salmonis* and harvested at 7 days post-infection, and *P. salmonis* grown to exponential phase in broth. Total RNA was extracted from each condition and subjected to RNA-seq or dual RNA-seq, as appropriate. Libraries were sequenced using paired-end Illumina technology, and differential gene expression was analyzed separately for host and pathogen.

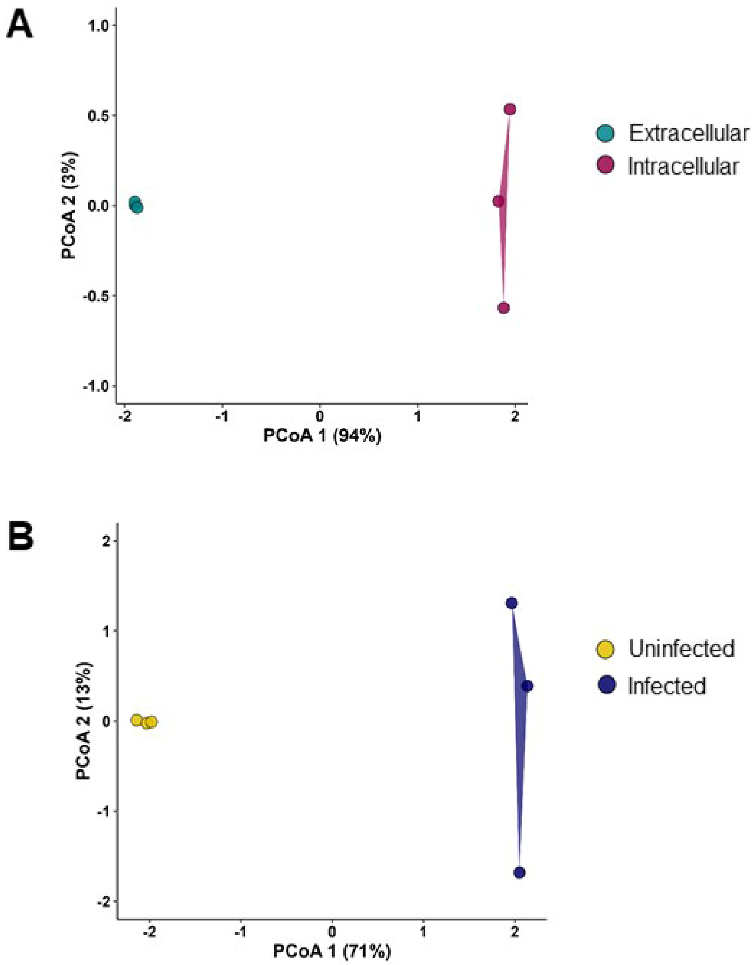

Fig S2. Principal-component analysis (PCA) of (A) the *P. salmonis* transcriptome of extracellular and intracellular bacteria, and (B) the transcriptome of *P. salmonis*-infected and uninfected SHK-1 cells.

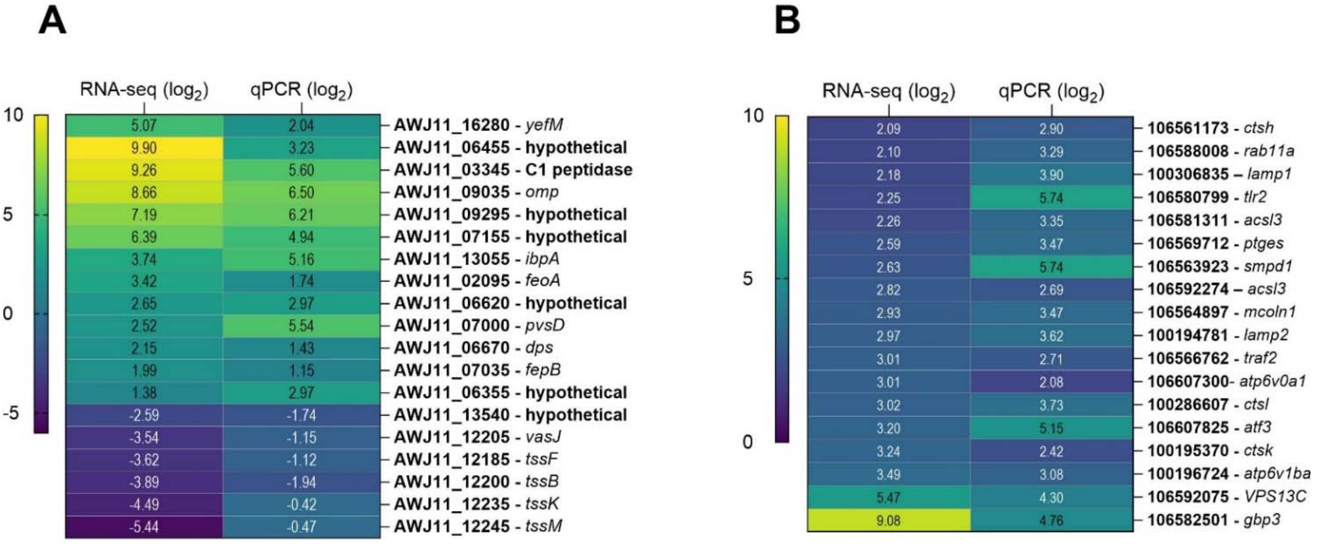

Fig. S3. (A) Heatmap showing fold-change values of selected differentially expressed *P. salmonis* genes comparing intracellular bacteria with bacteria grown in broth, as determined by RNA-seq and validated by RT-qPCR. (B) Heatmap showing fold-change values of selected host genes in SHK-1 cells comparing infected versus uninfected conditions, based on RNA-seq data and RT-qPCR validation. Color scale represents relative expression levels (log_2 fold change).

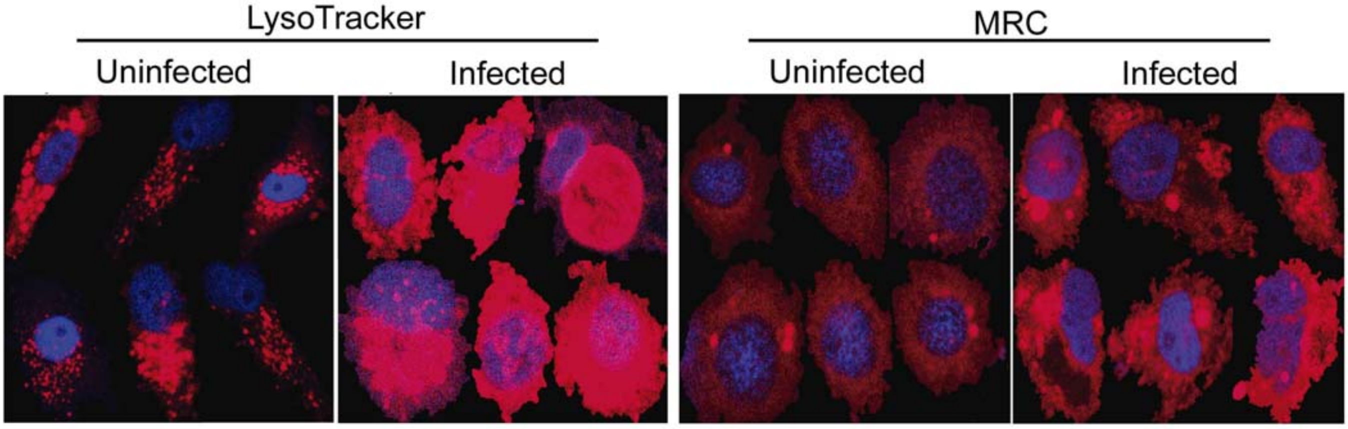

Fig. S4. Representative fluorescence images of lysosomal activity in SHK-1 cells. SHK-1 cells infected with *P. salmonis* or uninfected controls at 7 dpi were stained with LysoTracker Red DND-99 and Magic Red Cathepsin B (MRC). Each panel shows representative images from six independent cells.

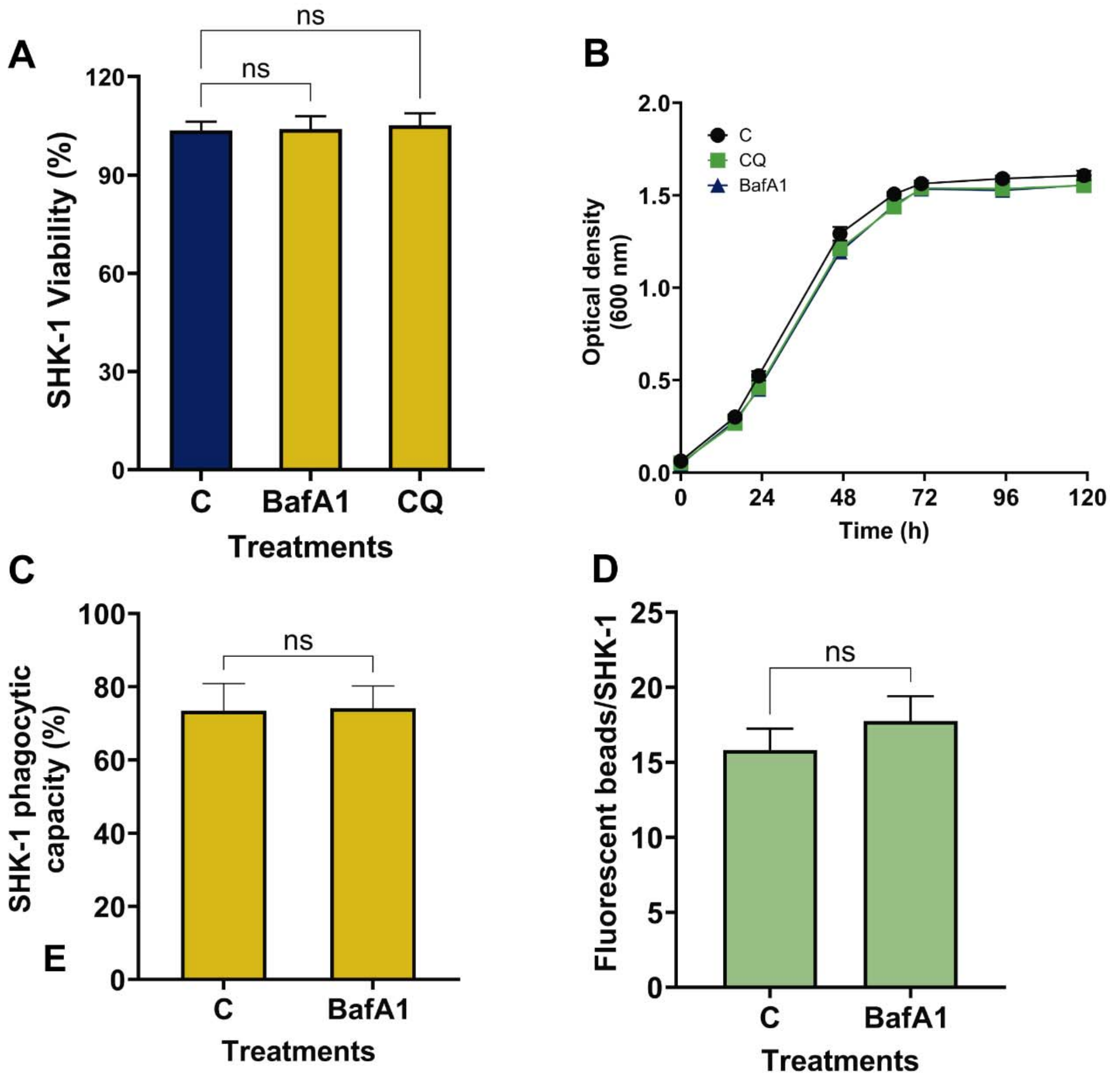

Fig S5. (A) Viability (%) of control (C), BafA1- or CQ-treated SHK-1 cells was assessed using the alamarBlue reagent. Statistical significance was assessed using ANOVA test, ns = no significant difference ($p < 0.05$, N = 3 biological replicates). (B) Growth curve of *P. salmonis* in NPB supplemented with 250 nM BafA1 or 25 µM of CQ. (C) Phagocytic capacity (%) of control SHK-1 cells (C) and cells treated with 250 nM BafA1 (BafA1) for 48 h. The percentage of phagocytic cells was calculated by counting the number of cells with FluoSpheres divided by the total number of cells present in the same field of view. (D) Average number of microspheres per cell in control SHK-1 cells (C) and in cells treated with 250 nM BafA1 (BafA1) for 48 h. In panels C-D, data reflect means ± SD (N = 3 biological replicates). Statistical significance was assessed using a t-test, ns = no significant difference (p < 0.05).

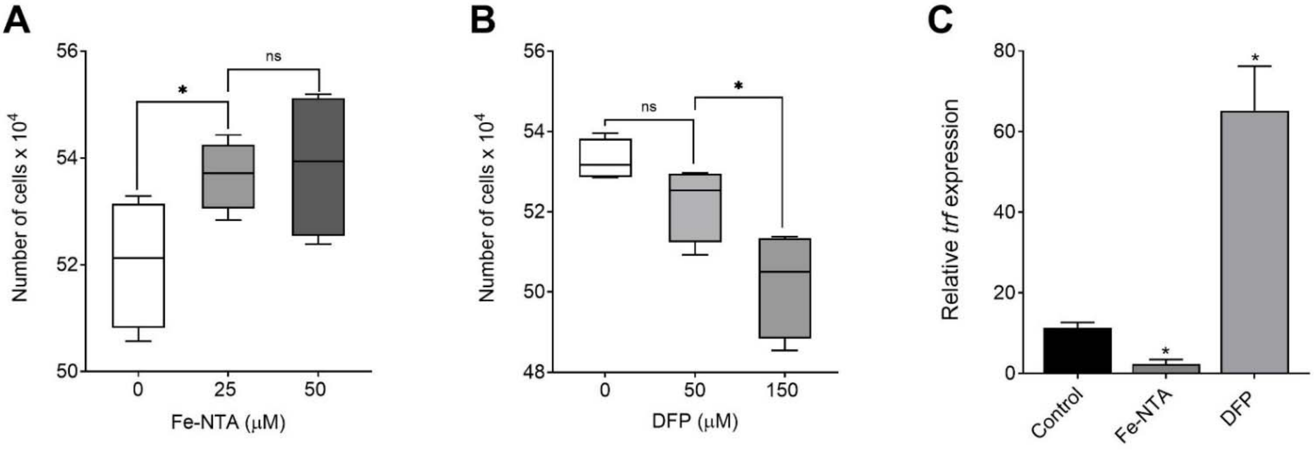

Fig S6. SHK-1 cells were cultured in L-15 medium supplemented with: (A) Fe-NTA (25 and 50 μM) or (B) deferiprone (DFP; 50 and 100 μM) (B) for 10 days. Cells were then washed with PBS, recovered by trypsinization, and stained with Trypan Blue. Cell numbers were determined using a Neubauer chamber. (C) Differential expression of *tfr* in infected and uninfected SHK-1 cells was assessed by qPCR. Fold-change values were calculated using the ΔCt method. Data represent mean ± SD of three independent experiments. Statistical significance was determined by unpaired t-test; $p < 0.05$.

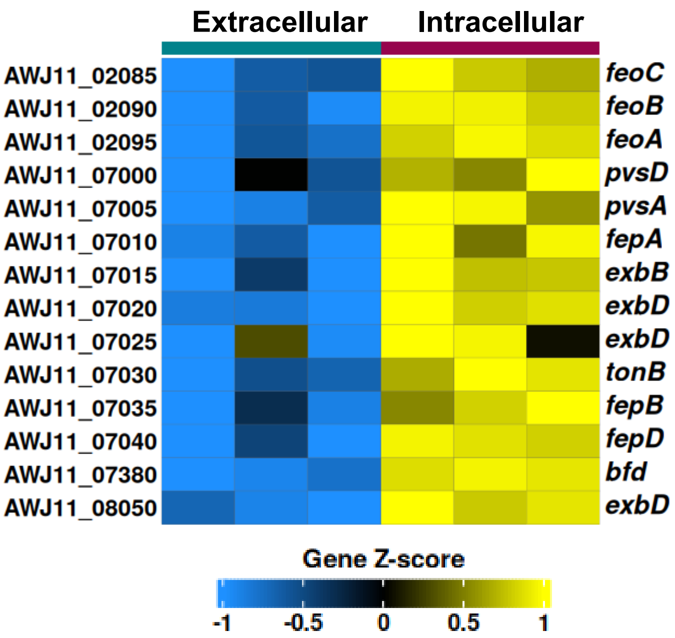

Fig. S7. Heatmap of expression values (z-score) for genes encoding iron uptake and storage proteins differentially expressed between intracellular and extracellular *P. salmonis* ($\log_2$ FC ≥ 2, adj. p value < 0.05).
