# Supplementary material for: Dual transcriptional analysis provides insights into the replicative niche of P. salmonis and the host response during infection
Source: mSystems. 2026 Apr 20;11(5):e00223-26. doi: 10.1128/msystems.00223-26 (PMC13185642; doi:10.1128/msystems.00223-26)
Supplement: Table S2 — Sequences of primers used for RT-qPCR. [file msystems.00223-26-s0003.pdf]

Table S2. Sequences of primers used for RT-qPCR

***P. salmonis* primers**				
**Gene ID**	**Gene name**		**Sequences**	
AWJ11_02095	*ferrous iron transport protein a*	*feoA*	Forward	TCTTTACAAGCTGGCACTTCC
			Reverse	GGCGGATACATAGGGCATAA
AWJ11_02755	*hypothetical protein//T4SE*		Forward	AGGTGAGGGAGTGTCTGGAA
			Reverse	AGGCTGTAACGCAGAGTCAG
AWJ11_03345	*C1 peptidase*		Forward	CCAATGATGCGTGGGGCTAA
			Reverse	TCTCAGCTTGTTGTTCCGGT
AWJ11_03780	*hypothetical protein//T4SE*		Forward	GCGACATCCATTCGGCAAAA
			Reverse	ACTTTTTCACCATCCGGGCA
AWJ11_03795	*hypothetical protein//T4SE*		Forward	TATTCCGCTTCCGCTTTGGA
			Reverse	CAAGGCAGTAGCAACGAGGA
AWJ11_04115	*hypothetical protein//T4SE*		Forward	GGTCTGCAACACTTGTGAAC
			Reverse	TCGGCAGCTCTACAAATTGC
AWJ11_04125	*hypothetical protein//T4SE*		Forward	AATAAGCTTTGCCGCCTCTG
			Reverse	CGGTGGGTGTCCTAGTAGTTC
AWJ11_04445	*hypothetical protein//T4SE*		Forward	TCGTTGGAGTGATTCTTTGGC
			Reverse	CACGAAACGCAGGCTTTCAA
AWJ11_05335	*hypothetical protein//T4SE*		Forward	CAAAAAGCACTCGCTGCCTC
			Reverse	GACCATAGCCGTGAAGCCAT
AWJ11_05370	*hypothetical protein//T4SE*		Forward	TGAAGCCCCTTGAGATTGGG
			Reverse	CCTCAGCAACACTCTGACGA
AWJ11_06410	*type iv secretion system dotg/icme*	*dotG/icmE*	Forward	GCAGCCAAAATCAGCAGAGG
			Reverse	CGTTGATGACGCAACCAATGT
AWJ11_06420	*type iv secretion system dote/icmc*	*dotE/icmC*	Forward	ATGGCTGCTCTATCAGGGGT
			Reverse	CCCGCCTTGACCAGAATGAC
AWJ11_06430	*type iv secretion system dotd/trah*	*dotD*	Forward	TTTCGGTGTTACCAGCAGTGA
			Reverse	TGAACCGCAGCAAATAACGC
AWJ11_06440	*type iv secretion system dotb*	*dotB*	Forward	GAACCATTACGGCAACACCG
			Reverse	GATCACAACCACACCTTGCG
AWJ11_06450	*type iv secretion system dotl/icmo*	*dotL/icmO*	Forward	AGGGTGGCGATGCTGATATG
			Reverse	GACGTACCGTTCCTGCATCA
AWJ11_06460	*type iv secretion system dotm/icmp*	*dotM/icmP*	Forward	CGTGACTGCTCGGGACTTAT
			Reverse	AACAATCGGCTGCGTTTCAG
AWJ11_06465	*type iv secretion system dotn/icmj*	*dotN/icmJ*	Forward	AAGCCTTGCCTGGGAATAACA
			Reverse	AGCTGTATGCCATCCCTGAC
AWJ11_06505		*icmV*	Forward	TGCTGCTTCTTTAGCTTGCTT

	type iv secretion system icmv		Reverse	TGGCCGACTTTAATCTGGGT
AWJ11_06620	*hypothetical protein*		Forward	TGTTCGTTGCTCTTCAGCCA
			Reverse	AAACCGCGATGAAAACCCAC
AWJ11_06670	*dna starvation/stationary phase protection protein*	*dps*	Forward	GCTGCTCACCCAAGGTTAAA
			Reverse	TGGAACATTAAGGGGCAACAA
AWJ11_07000	*siderophore biosynthesis protein*	*pvsD*	Forward	ACAGTGGTCTTGTGTGGAGT
			Reverse	GTGACAGCCCCCATTTGGTA
AWJ11_07035	*iron-siderophore abc transporter substrate-binding protein*	*fepB*	Forward	CGGTTAAAGTGCCGGTTAAG
			Reverse	CGCCCTACACTTTCAACTCC
AWJ11_07155	*hypothetical protein//T4SE*		Forward	TCCAGCAGAATCGAGGTTGT
			Reverse	TGGACCAACCCTTCAGGACT
AWJ11_08225	*protein kinase//T4SE*		Forward	TGGAAGAAGGAGCGGGAAAC
			Reverse	GATTTTGACGCCACACCAGC
AWJ11_09035	*outer membrane beta-barrel protein y1260*	*ompA*	Forward	GCGGAATGGATACAGCCCAG
			Reverse	CGACCAGCAAAACCACGTAAG
AWJ11_09295	*hypothetical protein//T4SE*		Forward	GGAAAGGCTGCATTCGCTAC
			Reverse	AGGTGCGGATTTGAGAGGAG
AWJ11_09705	*response regulator*	*irpR*	Forward	CTCTCTCGCCGTGAGTTTGT
			Reverse	TTGCGCAGGTTGTGAATGTG
AWJ11_10425	*hypothetical protein//T4SE*		Forward	GCCAGCAGAAAACAGGCAAC
			Reverse	TTTGAGTCGCTTGAAACCGC
AWJ11_12185	*type vi secretion system baseplate subunit tssf*	*tssF*	Forward	TGGCTGTGGGCGAATAAACT
			Reverse	AAAGTGTGATGGGGCGTGAT
AWJ11_12200	*type vi secretion system contractile sheath small subunit*	*tssB*	Forward	GCCCTCTTGCTTTTCTATACCC
			Reverse	CCACTTGCAAATGTGCCTGA
AWJ11_12205	*type vi secretion system vasl-like protein*	*vasL*	Forward	ACTCGACGGCATCAGGGTTA
			Reverse	ATGAATCCAGTCGTTGAGCCTT
AWJ11_12235	*type vi secretion system baseplate subunit tssk*	*tssK*	Forward	AGGTAACCCCGCCATTCAAA
			Reverse	GCTATTGTCAGCAAAGCGTGT
AWJ11_12245	*type vi secretion system membrane subunit tssm*	*tssM*	Forward	ACCAAGGCGATGACACAGTT
			Reverse	CCAGATGGAGGCAATGGTGA
AWJ11_13055	*heat-shock protein ibpa*	*ibpA*	Forward	GCCTCTGGAATCTCACGCAT
			Reverse	AAAGGTATTGCTGAGCGTGC
AWJ11_11105	*serine hydroxymethyltransferase*	*glyA*	Forward	GACTCGCGTACCATTGCAGA
			Reverse	GCACACGCGGACTCGTATAA

AWJ11_15880	*recombinational dna repair atpase recf*	*recf*	Forward	AAAATTTTGTTTAGCAATATTAGCGTTGTTTTT
			Reverse	ACTCTTATTTTCATCCCAACCAGCAT
AWJ11_15880	*recombinational dna repair atpase recf*	*recf*	Probe	FAM-ACGCCCAATTTTCA-NFQ
			Forward	CGCCTTCAAGCCAATTGTGG
			Reverse	GCAAGCTTTTCACCTTGCCA

**SHK-1 cell primers**				
**Gene ID**	***Gene name***		**Strand**	**Primer sequence**
100194781	*lysosomal-associated membrane protein 2*	*lamp-2*	Forward	GGTCGTAGGAAGACCTATGTGG
			Reverse	TTGAAAGACTGCACTGAGAGCC
106564897	*mucolipin-1*	*mcoln1*	Forward	TGACACGGCAAACAGGGTAA
			Reverse	TCACTACGGGTCCCCTTTCA
106563923	*sphingomyelin phosphodiesterase 1*	*smpd1*	Forward	TATGACGAGGCGACGATGAC
			Reverse	AACAAACCGAGAACTGCCCT
106607300	*V-type proton ATPase 116 kDa subunit a1-like/VATpase*	*atp6v0a1*	Forward	CTTCGACGAGGTCAACTCCA
			Reverse	CAGGGTCCTCCAGTGAGTCT
100196724	*ATPase, H+ transporting, lysosomal, V1 subunit B, member a*	*atp6v1ba*	Forward	TGTGATGGACTACAGCGACG
			Reverse	CGCTCAATAGTGGGGTCGTT
106579135	*V-type proton ATPase 116 kDa subunit a1*	*vATPaseB*	Forward	CAACTCAAGTGTGGCTGACG
			Reverse	AACAGTTCCCCCATGGTGAC
100195370	*Cathepsin K*	*catk*	Forward	ATAGACCTCAGCCCCCAGAA
			Reverse	GGTAAGCCTCCTCTGTGTCG
100286607	*Cathepsin L1*	*catl*	Forward	ACCAGGGATCATGTGGGTCT
			Reverse	TGCCAGTCTTCCTGAACTGC
100306835	*lysosomal-associated membrane protein 1*	*lamp1*	Forward	GCCAATGCCACAAACCTCAG
			Reverse	CAGACCAAGCTGCTACCACA
106592075	*vacuolar protein sorting-associated protein 13C-like vPSAP13c*	*vPSAP13C*	Forward	TGTTACCGCTGCCCAGAATAAT
			Reverse	CAGGAACAGTGTACAGGCTGAT
106588008	*ras-related protein Rab-11A*	*rab11a*	Forward	TCGTCGCATTTTAAACAGCTT
			Reverse	GGTGAAACGAGAGAGCAGGT
106561173	*cathepsin H*	*cath*	Forward	AGTCAGTCACAGCAATCGCA
			Reverse	CAAATGCTTGACTGGGGAGC
106592274	*fatty acid CoA ligase Acsl3-like*	*acsl3*	Forward	ACAATAGCCTAGCCCTGAAGA
			Reverse	AGAAAGCATGACCTACCTGTCC
106563923	*sphingomyelin phosphodiesterase 1*	*smpd1*	Forward	TATGACGAGGCGACGATGAC
			Reverse	AACAAACCGAGAACTGCCCT

106569712	*prostaglandin E synthase*	*ptges*	Forward	TTGTGGCTCTCGGCTGTAAG
			Reverse	GCAGGAGGATGTGCTTGCTA
106581311	*fatty acid CoA ligase Acsl3*	*acsl3*	Forward	TCCTATGCTGAGACCCACAAG
			Reverse	CAGGGTAGAGTACAGAGTGACC
106580799	*toll-like receptor 2 type-2*	*tlr2*	Forward	TGAATAACATGAGCCACCCACC
			Reverse	AATCCCACTTTGTAACACAACACC
106582501	*guanylate-binding protein 3*	*gbp3*	Forward	TGTGTGGACCGTCAGAGATT
			Reverse	CATAACGCTACGCTTTGCATC
106607825	*cyclic AMP-dependent transcription factor ATF-3*	*batf*	Forward	GCCTTCTGTCAGGCCATCAA
			Reverse	TGACATTCGCCCTATGTGCC
106566762	*tnf receptor-associated factor 2b*	*traf2*	Forward	AACTGTTGTGTGTGTGCCTTAT
			Reverse	AGGGAGGAGTCTAAAGAGGGC
106588478	*elongation factor 1-alpha*	*elf1a*	Forward	CACCACCGGCCATCTGATCTACAA
			Reverse	TCAGCAGCCTCCTTCTCGAACTTC
